# A novel pathway for stemness propagation and chemoresistance in non-small cell lung cancer via phosphorylated PKM2-loaded small extracellular vesicles

**DOI:** 10.7150/thno.103722

**Published:** 2025-02-24

**Authors:** Jingyi Wang, Liu Liu, Xinyu Gao, Xiyu Liu, Yitian Dai, Zijun Mao, Shengzhe Huang, Junjian Li, Dongliang Wang, Yu Qi, Yingwen Han, Yunjing Xu, Corrine Ying Xuan Chua, Alessandro Grattoni, Wenhui Xie, Hao Yang, Gang Huang

**Affiliations:** 1 Department of Nuclear Medicine, Shanghai Chest Hospital, Shanghai Jiao Tong University School of Medicine, Shanghai 200025, China.; 2 Shanghai Key Laboratory of Molecular Imaging, Jiading District Central Hospital Affiliated Shanghai University of Medicine and Health Sciences, Shanghai 201318, China.; 3 Department of Nanomedicine, Houston Methodist Research Institute, Houston, TX 77030, USA.; 4 Laboratory of Stem Cell Biology and Engineering, New York Blood Center, New York, NY 10065, USA.; 5 Department of Oncology, Shanghai General Hospital, Shanghai Jiao Tong University School of Medicine, Shanghai 200080, China.; 6 Department of Nuclear Medicine, Cancer Institute, Fudan University Shanghai Cancer Center, Shanghai 200032, China.; 7 Department of Pathology, Fudan University Shanghai Cancer Center, Shanghai, 200032, China.; 8 Department of Surgery, Houston Methodist Hospital, Houston, TX 77030, USA.; 9 Department of Radiation Oncology, Houston Methodist Hospital, Houston, TX 77030, USA.

**Keywords:** cancer stem cells, small extracellular vesicles, pY105-PKM2, IQGAP1, drug resistance

## Abstract

**Rationale:** Non-small cell lung cancer (NSCLC) is a predominant cause of cancer-related mortality, with its progression and treatment resistance significantly influenced by cancer stem cells (CSCs) and their complex intercellular communication mechanisms. Small extracellular vesicles (sEVs) have emerged as pivotal mediators of intercellular signaling, affecting tumor microenvironment modulation and therapeutic resistance. This study investigates the role of CSC-derived sEVs in transmitting stemness traits through the selective sorting of pyruvate kinase M2 phosphorylated at the Y105 site (pY105-PKM2), mediated by the adaptor protein IQGAP1, which supports CSC maintenance and drug resistance in NSCLC.

**Methods:** *In vitro* and *in vivo* experiments, including proteomic and transcriptomic analyses, were conducted to identify key regulators of sEV-mediated signaling. Immunoprecipitation, proximity ligation assays, and immunofluorescence were used to examine the role of IQGAP1 in the sorting of pY105-PKM2 into sEVs. Functional assays, including sphere formation, chemoresistance tests, metabolic assessments, and cell cycle analysis, were conducted to evaluate the effects of sEV-mediated delivery of pY105-PKM2 on recipient cells. Additionally, immunohistochemistry and survival analysis were performed on tumor samples from NSCLC patients to establish clinical correlations.

**Results**: We unveiled a novel mechanism by which CSC-derived sEVs transmit stemness traits to replenish the CSC pool in NSCLC. CSC-derived sEVs were enriched with pY105-PKM2, correlating with enhanced stemness, chemoresistance, and poor clinical outcomes. Mechanistically, IQGAP1 was identified as an adaptor facilitating the selective sorting of pY105-PKM2 into sEVs through interactions with the ESCRT component TSG101. Recipient cells treated with CSC-derived sEVs exhibited metabolic reprogramming, slower cell cycle progression, and enhanced chemoresistance. The synergistic role of IQGAP1 and pY105-PKM2 was confirmed, highlighting their critical contributions to CSC maintenance and malignant progression.

**Conclusion:** This study highlights the critical role of CSC-derived sEVs in NSCLC progression and therapy resistance through the IQGAP1-mediated selective sorting of pY105-PKM2. By uncovering this novel pathway, our findings provide valuable insights into CSC pool replenishment and therapeutic resistance mechanisms in NSCLC, identifying IQGAP1 and pY105-PKM2 as promising therapeutic targets for mitigating CSC-driven malignancy and enhancing treatment efficacy.

## Introduction

Lung cancer ranks as one of the most lethal types of cancer worldwide, with non-small cell lung cancer (NSCLC) being the predominant subtype. According to the latest GLOBOCAN 2020 data, lung cancer has an incidence rate of 11.4% among all malignancies globally, placing it second, and a mortality rate of 18%, ranking it first 1. Despite advancements in treatment, including the use of platinum-based drugs and docetaxel, therapeutic resistance continues to hinder long-term efficacy 2. Recent strategies combining targeted therapy and immunotherapy with chemotherapy have yielded short-term benefits for some patients 3-5. However, overcoming the inevitable issue of drug resistance in NSCLC remains a global challenge and priority.

Recent studies have highlighted the critical role of cancer stem cells (CSCs) in tumor initiation, progression, and therapeutic resistance 6-8. CSCs are characterized by their capacity for self-renewal and differentiation into diverse cell lineages within the tumor mass, thereby contributing to the complexity and treatment-resistant nature of the disease 9. Traditional strategies focused on inhibiting CSC-specific surface markers, such as CD44 or CD133, or the disruption of their signaling pathways, including Hedgehog, Wnt, and Notch, have largely failed to overcome therapeutic resistance 10. Emerging evidence has illuminated a fundamental challenge: the hierarchy within CSC populations is not hardwired. Douglas Hanahan's recent proposition of “phenotypic plasticity” as a novel dimension enhances our comprehension of cancer's hallmarks by highlighting the potential of cancer cells to dedifferentiate to a progenitor-like state from a fully differentiated one 11, 12. Cellular plasticity within the tumor microenvironment allows for the reprogramming of non-CSCs or differentiated cancer cells back into CSCs, suggesting a dynamic equilibrium that permits the replenishment of the CSC pool, thereby facilitating tumor recurrence and resistance to therapy 13, 14. Therefore, elucidating the mechanisms behind CSC pool replenishment is essential for completely eradicating CSCs and may hold the key to more effective cancer treatments.

Small extracellular vesicles (sEVs), as defined by the International Society for Extracellular Vesicles, are entities less than 200 nm in diameter, generated through the amalgamation of multivesicular bodies with the plasma membrane 15. These vesicles, ubiquitously released by a wide range of cells and found in various bodily fluids, play a critical role in cellular communication, carrying an array of molecules, including proteins, lipids, RNA, and DNA 16-18. Clinical observations have documented more sEVs in cancerous cells relative to their benign counterparts, implicating them in various pathological processes, such as angiogenesis, chemoresistance, cellular differentiation within the tumor microenvironment, immune modulation, and pre-metastatic niche regulation 19, 20. Recent studies have also highlighted the role of CSC-derived sEVs in immune evasion, where they modulate the tumor microenvironment to suppress antitumor immune responses, facilitating immune escape and promoting tumor progression 21. While sEVs are recognized for their role in transferring bioactive molecules that contribute to the aggressive behavior of cancer, the specific pathways and molecular cargos driving CSC maintenance and expansion, particularly in NSCLC, remain underexplored.

Another facet of oncology that has attracted interest is the altered metabolism within cancer cells, serving as a foundation for innovative therapeutic interventions 11, 22. Previous research in this domain has not adequately addressed the metabolic heterogeneity within cancer cell populations 23. The distinct metabolic behavior of CSCs represents another layer of complexity in cancer 24-26. Our research has demonstrated that PKM2 cargo in sEVs drives cisplatin resistance 27. However, it remains uncertain whether this phenomenon is associated with CSC behavior. Recent findings have suggested a correlation between the levels of pyruvate kinase M2 phosphorylated at the Y105 site (pY105-PKM2) and the emergence of stemness features in breast cancer cells, positing an association between metabolic shifts and the acquisition of stem cell-like traits in cancer 28. Nevertheless, the specific role of pY105-PKM2 in promoting a CSC-like phenotype in NSCLC cells and its integration into the regulatory networks associated with sEVs needs to be clearly defined.

In this study, we investigated the role of CSC-derived sEVs in NSCLC progression and chemoresistance, focusing on the selective incorporation of pY105-PKM2. We identified IQGAP1 as a key mediator of this process, linking pY105-PKM2 to TSG101 and facilitating its packaging into sEVs. These sEVs enhance stemness, metabolic reprogramming, and drug resistance in recipient cells. Additionally, we confirmed the synergistic role of IQGAP1 and pY105-PKM2, highlighting their critical contributions to CSC maintenance and malignant progression. Our findings provide new insights into CSC maintenance and sEV-mediated resistance, identifying potential therapeutic targets to disrupt CSC-driven malignancy.

## Materials and methods

### Cell culture

Human lung cancer cell line A549 (ATCC, Cat# CCL-185) was cultured in RPMI-1640 medium supplemented with 10% fetal bovine serum (Yeasen Biotechnology, Shanghai, China) and 1% penicillin-streptomycin (Gibco, Cat# 15140122, MA, USA). Cisplatin-resistant A549 cells (A549CR) were developed by gradually increasing the cisplatin concentration over 10 months, with a final maintenance dose of 1.0 μg/mL cisplatin.

For sphere formation, cells in the exponential growth phase were plated at a density of 1,000 cells/mL in serum-free DMEM/F12 medium (Absin Bioscience Inc., Shanghai, China) supplemented with B-27 (1:50, Absin Bioscience Inc.), N2 supplement (1:100, Absin Bioscience Inc.), and human recombinant epidermal growth factor (20 ng/mL, Sigma-Aldrich, Darmstadt, Germany). Cells were cultured in ultra-low attachment 6-well plates (Corning, Cat# 3471, NY, USA) for 7-14 days to form spheres, which were identified using a phase-contrast microscope. Spheres exceeding 50 µm in diameter were quantified.

All cell lines were authenticated by STR profiling and tested for mycoplasma contamination every 2 weeks. Cells were maintained at 37 °C in a humidified atmosphere with 5% CO₂. Paclitaxel, cisplatin, TEPP-46, and Apcin were obtained from Selleck Chemicals (TX, USA).

### Cell line establishment

To investigate the role of PKM2 phosphorylation at Y105 and IQGAP1 in NSCLC, we established multiple genetically modified cell lines. Stable PKM2^WT^-expressing and PKM2^Y105F^-expressing A549 cells were generated by CRISPR-Cas9-mediated PKM2 knockout, followed by lentiviral transduction with plasmids encoding either PKM2^WT^ or the non-phosphorylatable PKM2^Y105F^ mutant. Selection was performed using puromycin (Selleck Chemicals). Two guide RNA sequences were used for PKM2 knockout and for control: CTTGCCTGCTGTGTCGGAGAAGG and GCAAAATCGAGAATCATGAGGGG. The pLenti-Flag-PKM2^Y105F^ plasmid was constructed by amplifying Flag-PKM2^Y105F^ mutant DNA with primers PKM2-PrimerF and PKM2-PrimerR and cloning into the pLenti vector.

For IQGAP1 silencing, A549-PKM2^WT^ and A549-PKM2^Y105F^ cells were transfected with 20 nM siRNA using Lipofectamine RNAiMAX (Thermo Fisher, USA) following the manufacturer's protocol. Cells were seeded at 1.5 × 10⁵ cells per well in six-well plates and transfected at 50-60% confluency. siRNA and Lipofectamine RNAiMAX were separately diluted in Opti-MEM, incubated for 5 min, mixed, and left for 20 min at room temperature to form transfection complexes. The mixture was then added to antibiotic-free RPMI-1640 medium, and cells were incubated for 48 h before further analysis. The sequences of the siRNAs used for IQGAP1 silencing were:

siRNA-IQGAP1#1: GCCCACUUAAGCAUCAUUATT/UAAUGAUGCUUAAGUGGGCTT; siRNA-IQGAP1#2: CCAGUCGUGAAGGAAAUUATT/UAAUUUCCUUCACGACUGGTT; siRNA-IQGAP1#3: GUUGCAGUCAUGAAAUUAUTT/AUAAUUUCAUGACUGCAACTT; siRNA-IQGAP1#4: GCGACAAAGUCCUGAACAUTT/AUGUUCAGGACUUUGUCGCTT.

To further assess the role of IQGAP1 in CSC maintenance and chemoresistance, we established IQGAP1-overexpressing A549 cells, as well as A549 cell lines co-expressing IQGAP1 with PKM2^WT^ or PKM2^Y105F^. These cell lines were generated via lentiviral transduction and selected using puromycin. The expression of IQGAP1 and PKM2 variants was validated by fluorescent protein expression, where IQGAP1 was tagged with red fluorescence, and PKM2^WT^ and PKM2^Y105F^ were tagged with green fluorescence, before further experiments.

### Colony formation assay

Clonogenic potential was assessed using a colony formation assay. A549, A549CR, and stable PKM2^WT^- or PKM2^Y105F^-expressing cells were seeded in 6-well plates at 1,000 cells/well and maintained in complete growth medium for 10 days. Colonies were fixed with 4% paraformaldehyde and stained with crystal violet. Images of stained colonies were captured using a digital camera.

### CSC enrichment

CSCs were isolated from A549 tumors extracted from tumor-bearing mice. Tumors were first minced into small fragments using sterile scissors, and the minced tissue was then digested in a solution comprising 100 mg/mL collagenase type I (Sigma-Aldrich, Cat# C0130, TX, USA), 3 mM CaCl2 (Sigma-Aldrich, Cat# C1016), and 10 mg/mL hyaluronidase (Sigma-Aldrich, Cat# H3506) at 37 °C for 4 h with gentle agitation. Post-digestion, enzymatic activity was halted, and the resulting cell suspension was filtered through a 40 µm cell strainer (Corning, Cat# 431750) to eliminate debris and undigested tissue fragments. The single-cell suspension obtained was further purified and cultured in serum-free DMEM/F12 medium, supplemented with B-27, N2 supplement, and human recombinant epidermal growth factor in ultra-low attachment 6-well plates, to enrich for CSCs, as described before.

### Flow cytometry assay

CD133^+^ and CD44^+^ cell populations were quantified using flow cytometry (NovoCyte, Agilent Technologies, CA, USA). Cells were detached with trypsin-EDTA (ThermoFisher, MA, USA), washed, and suspended in phosphate-buffered saline (PBS). Anti-CD133-APC (10 μL, Miltenyi Biotec, Cat# 130-113-668, CA, USA) and anti-CD44-PE (10 μL, Miltenyi Biotec, Cat# 130-113-342) antibodies were added to 100 μL of cell suspension. Non-stained cells served as controls. Following incubation in the dark at 4 °C for 15 min and two PBS washes, cells were resuspended in 300 μL of PBS and kept on ice. Data were acquired on a flow cytometer and analyzed using NovoExpress software (version 1.5.0, Agilent Technologies).

### Apoptosis assay

Apoptosis was measured after a 24 h exposure to 5 μg/mL cisplatin or 200 ng/mL paclitaxel using the Annexin V-PE/7-AAD Apoptosis Detection Kit (Yeasen Biotechnology). Cells were collected, washed with cold PBS, and centrifuged at 300 g for 5 min. Cells were resuspended in 1× binding buffer to a final density of 1 × 10^6^ cells/mL. Annexin V/PE (5 μL) and 7-AAD (10 μL) were added to 100 μL of cell suspension, incubated in the dark at room temperature for 15 min, and analyzed by flow cytometry (NovoCyte, Agilent Technologies) with data processed using NovoExpress software.

### Cytotoxicity assays

Cell viability in response to cisplatin and paclitaxel was assessed using the Cell Counting Kit-8 (Yeasen Biotechnology). Cells were seeded in 96-well plates at 5,000 cells/well and exposed to various drug concentrations for 48 h. Optical density at 450 nm was measured, and IC50 values were calculated by generating dose-response curves.

### Liquid chromatography-tandem mass spectrometry (LC-MS/MS)

Proteomic analysis was performed using LC-MS/MS. Cell lysates were prepared in RIPA buffer, protein concentrations were determined using the BCA protein assay, and binding partners of Flag-tagged proteins were immunoprecipitated. Trypsin-digested peptides were analyzed on a Q Exactive HF-X Hybrid Quadrupole-Orbitrap Mass Spectrometer with an Ultimate 3000 RSLCnano System. Data analysis was conducted using Proteome Discoverer 2.4 with SEQUEST HT searching against the Human UniProt database and validated using Percolator at a 1% FDR.

### Isolation and characterization of sEVs

Cells were cultured in serum-free DMEM or DMEM/F12 for 72 h. The medium was collected, centrifuged at 2,000 g and 10,000 g for 10 min each, filtered through a 0.45 μm filter (MilliporeSigma, Darmstadt, Germany), and subjected to ultracentrifugation at 120,000 g for 90 min using an Optima XPN-100 centrifuge (Beckman Coulter, CA, USA). The pellet was resuspended in PBS, re-centrifuged, and stored at -80 °C. sEV concentration and size distribution were analyzed using nanoparticle tracking analysis (ZetaView, Particle Metrix, Meerbusch, Germany) and nanoflow cytometry. sEV morphology was assessed using transmission electron microscopy (FEI Tecnai G2 Spirit, OR, USA), and protein markers were evaluated by western blot.

### Fluorescence labeling and cellular uptake of sEVs

For *in vitro* uptake analysis, sEVs were labeled with PKH67 dye (PKH67GL, Sigma-Aldrich, MO, USA) following the manufacturer's instructions. Labeled sEVs were centrifuged to remove excess dye, resuspended in PBS, and added to lung cancer cells plated on coverslips. After 24 h of incubation, cells were fixed, and stained with DAPI (Yeasen Biotechnology, Cat# 40728ES03) to visualize nuclei and phalloidin (Yeasen Biotechnology, Cat# 40762ES75) to stain the cytoskeleton. Imaging was performed using a confocal microscope (TCS-SP8, Leica, Heidelberg, Germany).

For *in vivo* tracking of sEV uptake by tumors, sEVs were labeled with the lipophilic near-infrared dye Dir (MedchemExpress, Cat# HY-D1048, NJ, USA). Briefly, sEVs were incubated with 1 μM Dir dye at room temperature for 15 min in the dark, followed by ultracentrifugation at 120,000 g for 70 min to remove unbound dye. The labeled sEVs were resuspended in PBS and injected intravenously into tumor-bearing nude mice. After two injections, fluorescence signals were detected using IVIS Spectrum imaging (PerkinElmer, MA, USA) to assess sEV biodistribution and tumor uptake.

### Glucose uptake and lactate production assay

Cells were cultured in 12-well plates. After 24 h, the medium was replaced with 500 μL serum-free high-glucose DMEM for 8 h. The conditioned medium was then collected and glucose uptake and lactate production were measured using kits (Sigma-Aldrich, MO, USA, and Jiancheng Bioengineering Institute, Nanjing, China). The results were normalized to the total cell number.

### Seahorse assay

Cells were pre-treated with Vec-sEV, PKM2^WT^-sEV, or PKM2^Y105F^-sEV for 48 h and seeded in seahorse XF96 microplates at 2 × 10⁴ cells per well and cultured for 24 h. The medium was replaced with seahorse XF base medium (pH 7.4) supplemented with 2 mM glutamine, 25 mM glucose, and 1 mM sodium pyruvate, followed by incubation in a CO₂-free incubator for 1 h. For the mitochondrial stress test, 1.5 μM oligomycin, 1.0 μM FCCP, and 0.5 μM rotenone were sequentially injected to assess basal respiration, ATP production, maximal respiration, and spare respiratory capacity. Oxygen consumption rate (OCR) was recorded using the Seahorse XF96 Analyzer (Agilent Technologies, CA, USA).

### RNA-sequencing

Transcriptomic sequencing was performed on A549 cells treated with sEVs derived from A549-PKM2^WT^ and A549-PKM2^Y105F^cells. Total RNA was extracted using Trizol (Invitrogen, CA, USA), and quality was assessed using an Agilent 2100 Bioanalyzer. mRNA was enriched, fragmented, and converted into cDNA using the NEBNext Ultra RNA Library Prep Kit, followed by sequencing on an Illumina NovaSeq 6000. For miRNA profiling, sEVs derived from A549-PKM2^WT^ and A549-PKM2^Y105F^cells were subjected to small RNA sequencing. RNA was extracted using the miRNeasy Mini Kit (Qiagen, Hilden, Germany), and small RNA libraries were prepared with the NEBNext Multiplex Small RNA Library Prep Set. Libraries were sequenced on an Illumina NovaSeq 6000 platform. Bioinformatic analyses for both datasets, including differential expression and pathway enrichment, were conducted using Omicsmart, a real-time interactive platform for multi-omics analysis.

### Cell cycle assay

Cell cycle analysis was performed by fixing cells in 85% ethanol overnight at 4 °C. Cells were washed and resuspended in propidium iodide staining solution (500 μL, BD Biosciences, NJ, USA) for 15 min in the dark. DNA content was measured using flow cytometry (NovoCyte, Agilent Technologies) and analyzed using NovoExpress software.

### Immunoprecipitation and immunoblotting

Cell lysates were prepared using RIPA buffer (Epizyme, Shanghai, China). For immunoprecipitation, anti-Flag immunomagnetic beads (Epizyme, Cat# YJ007), anti-TSG101 (Proteintech, Cat# 67381-1, IL, USA), or anti-IQGAP1 (Cell Signaling Technology, Cat# 20648, MA, USA) antibodies were used with Protein A/G beads (Bimake, Cat# B23201, TX, USA). Proteins were separated by SDS-PAGE, transferred to PVDF membranes, and probed with primary antibodies: PKM2 (Cat# 3198S), phospho-PKM2 (Tyr105) (Cat# 3827), and IQGAP1 (Cat# 20648) from Cell Signaling Technology; NANOG (Cat# 14295-1-AP), OCT4 (Cat# 11263-1-AP), SOX2 (Cat# 11064-1-AP), Calnexin (Cat# 10427-2-AP ), CD44 ( Cat#60224-1-Ig), CD133 (Cat# 18470-1-AP), and TSG101 (Cat# 67381-1) from Proteintech; CDK1 (phospho T14 + Y15) (Cat# ab277772) from Abcam (MA, USA); JAK3 (Cat# CY7016) and ITK (Cat# CY6933) from Abways (Shanghai, China); Yes1 (Cat# R26126), Src (Cat# R25792), AXL (Cat# R23576), FAK (Cat# R24277), Cyclin B1 (Cat# R23324), CDK1 (Cat# R23884), and CDC25B (Cat# R381486) from Zen Bioscience (Chengdu, China); β-actin (Cat# AC206) from ABclonal (MA, USA); and FLAG (Cat# LF304) from Epizyme. Detection was performed using HRP-conjugated secondary antibodies and ECL reagent (Yeasen Biotechnology), and visualized with a chemiluminescence detection system (Tanon, Shanghai, China).

### Duo-link proximity ligation assay

The interactions between Flag-tagged proteins (PKM2^WT^ or PKM2^Y105F^) and TSG101 were assessed using the Duolink proximity ligation assay kit (orange, DUO92007, Sigma-Aldrich). Cells were fixed, permeabilized, blocked, and incubated with primary antibodies against Flag-tag and TSG101. Duolink probes were applied, followed by ligation and amplification. DAPI was used for counterstaining, and interactions were imaged using a confocal microscope (LSM 880, Zeiss, Oberkochen, Germany).

### Immunofluorescence

A549 cells expressing PKM2^WT^ or PKM2^Y105F^ and transfected with siRNA targeting IQGAP1 or a non-targeting siRNA control (siRNA-NC) were subjected to fixation, permeabilization, and blocking. The cells were incubated with primary antibodies against TSG101 (Proteintech, Cat# 14497-1-AP) and Flag (Epizyme, Cat# LF304), followed by secondary antibody incubation with anti-rabbit IgG conjugated with Alexa Fluor Cy3 (Yeasen Biotechnology, Cat# 33108ES60) and anti-mouse IgG conjugated with Alexa Fluor 647 (Yeasen Biotechnology, Cat# WA3323060). Nuclei were counterstained with DAPI, and fluorescent images were captured using a Leica TCS-SP8 confocal microscope.

### Tumor formation and *in vivo* limiting dilution assays

A549 cells expressing PKM2^WT^ or PKM2^Y105F^, infected with a luciferase-expressing adenovirus, were used for tumor formation assays. Cells (5 × 10^6^) were subcutaneously injected into 6-8-week-old male Balb/c mice (Shanghai Laboratory Animal Center). For limiting dilution assays, cells at varying concentrations were injected into bilateral flanks of mice, with tumor incidence assessed after three weeks via bioluminescence imaging. Tumorigenic capacity was calculated based on tumor formation frequency. To assess sEV-related tumorigenicity, 6-8-week-old male Balb/c mice were injected with 5 × 10^6^ luciferase-tagged A549 cells. Tumors were allowed to grow to ~100 mm³ before sEV injections (1 × 10^9^ particles) from different cell types were administered tri-weekly adjacent to the tumor site, and tumor volumes were measured. All animal procedures followed ethical guidelines from the Committee on Animal Research Ethics at the Shanghai Chest Hospital.

### Bioluminescence imaging of xenograft tumors in mice

Bioluminescence imaging was conducted using an IVIS Spectrum system (PerkinElmer) following intraperitoneal injection of D-luciferin (Yeasen Biotechnology). Mice were anesthetized with isoflurane during the imaging process to minimize movement and ensure consistent image capture. Imaging parameters were optimized to detect luminescence signal intensity.

### PET/CT imaging of xenograft tumors in mice

Mice were food-restricted for 6-8 h before PET/CT imaging to ensure optimal glucose uptake. Each mouse received an intravenous injection of 5 MBq of 18F-FDG. 30 min post-injection, mice were anesthetized with isoflurane and positioned in the Siemens Inveon PET/CT system (Siemens Medical Solutions, Erlangen, Germany), and PET/CT scans were performed to assess metabolic activity. PET images were reconstructed using a 3D-ordered subset expectation maximization algorithm, and CT images were used for anatomical reference and attenuation correction. Image analysis was performed using Inveon Research Workplace software (Siemens Medical Solutions).

### Immunohistochemical analysis

Xenograft tumor specimens were fixed, paraffin-embedded, and stained with hematoxylin and eosin. For immunohistochemistry, tissues were quenched with 3% H_2_O_2_ solution, blocked with 3% bovine serum albumin, and incubated with primary antibodies against PKM2 (Bioss, Cat#bs-0102M), phospho-PKM2 (Tyr105) (Bioss, Cat# bs-3334R,), OCT4 (Proteintech, Cat#, 11263-1-AP,), SOX2 (Proteintech, Cat# 11064-1-AP,), CDC25B (Zen Bioscience, Cat# R381486,), CyclinB1 (Cat# R23324, Zen Bioscience), and CDK1 (Zen Bioscience, Cat# R23884,). Sections were then incubated with HRP-conjugated goat anti-rabbit IgG secondary antibody (Proteintech, Cat# SA00001-2) and HRP-conjugated goat anti-rabbit IgG secondary antibody (Proteintech, Cat# SA00001-1) for 1 h at 37 °C. Signal visualization was performed using DAB substrate (ZSGB-BIO, Cat# ZLI-9018,) with reaction time controlled under microscopic observation. Nuclei were counterstained with hematoxylin, followed by dehydration, clearing, and mounting with neutral resin. Images were captured using a Nikon Eclipse E100 microscope equipped with a CCD camera (Nikon DS-Ri2).

Lung cancer tissues from patients at Shanghai Chest Hospital were used to create microarrays for immunohistochemistry of PKM2 (Bioss, Cat# bs-0102M), phospho-PKM2 (Tyr105) (Bioss, Cat# bs-3334R), CD133 (Abclonal, Cat# A0818), and IQGAP1 (Cell Signaling Technology, Cat# 20648). Immunohistochemistry scores were assessed independently by two pathologists. The proportion of positively stained cells was categorized as follows: 0 for 0-5%, 1 for 5-25%, 2 for 25-50%, and 3 for 50-100%. Staining intensity was graded as 0 (negative), 1 (weak expression), 2 (medium expression) and 3 (strong expression). The final immunohistochemistry score for each sample was determined by multiplying the positive cells rate score by the staining intensity score. An immunohistochemistry score < 6 is considered low expression, while a score > 6 is considered high expression. The ethical framework for the study was reviewed and approved by the Human Ethics Committee of Shanghai Chest Hospital.

### Statistical analysis

Data analysis was performed using GraphPad Prism 10 (GraphPad Software, Inc., USA) and SPSS 20.0 (SPSS, Inc., Chicago, IL). Data are expressed as mean ± SD. Student's t-test was used for analyzing data from colony and sphere formation assays. One-way ANOVA was used for independent variables with more than two group and followed by Tukey's post hoc test, as appropriate. Two-way ANOVA was used for tumor growth curves, followed by Bonferroni post hoc test. Statistical significance was defined as *p* < 0.05.

## Results

### CSC-derived sEVs drive stemness propagation and maintain the CSC pool

**Figure 1:**
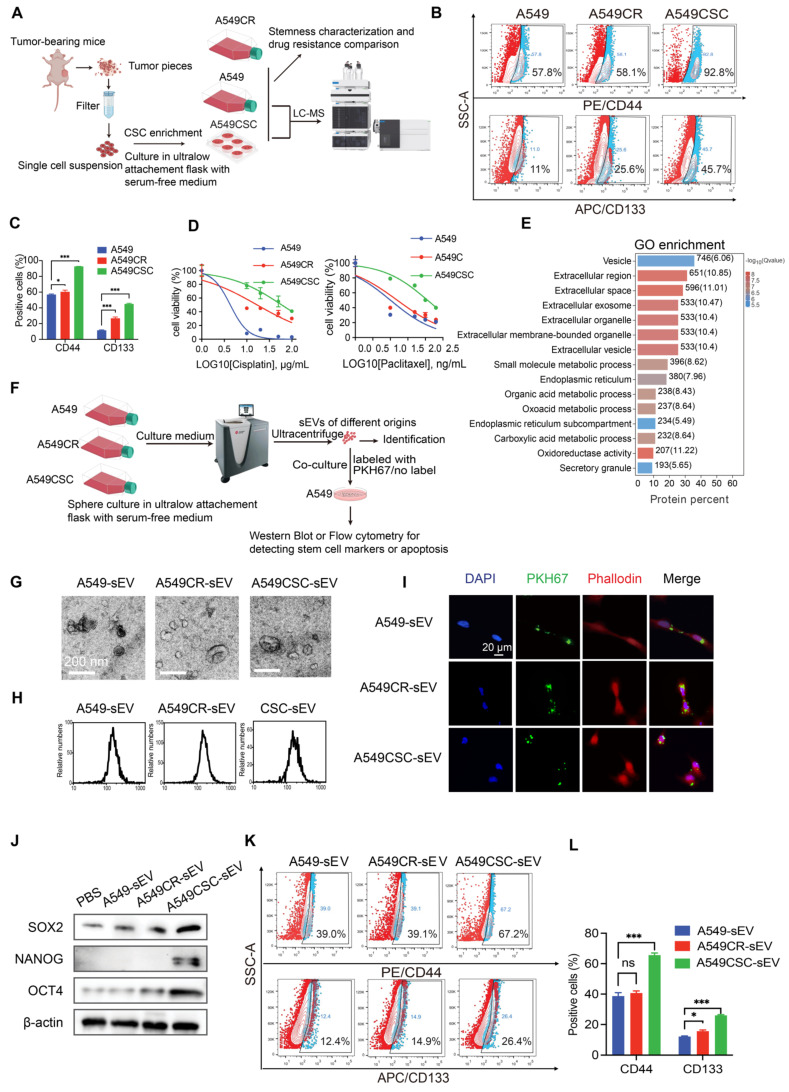
** Characterization of CSCs and their sEVs.** (A) Schematic representation of the workflow for CSC enrichment, characterization, and proteomic analysis. Tumor-bearing mice were used to generate single-cell suspensions, followed by sphere culture to enrich CSCs. Enriched CSCs were used for subsequent stemness characterization and drug resistance comparison. Proteomic profiling was conducted via LC-MS. (B) Flow cytometry analysis of CD44 and CD133 expression in A549, A549CR, and CSC-enriched populations. (C) Quantification of CD44- and CD133-positive cells across different populations. Data are presented as mean ± SD (*p < 0.05, ***p < 0.001, n = 3). Comparisons between groups were analyzed using one-way ANOVA, followed by Tukey's post-hoc test. (D) Dose-response curves for cisplatin and paclitaxel treatment in A549, A549CR, and CSCs. Data are presented as mean ± SD (n = 3). (E) GO enrichment analysis of proteins identified from proteomic profiling, focusing on extracellular vesicle-related pathways. (F) Diagram showing the isolation of sEVs from different cell populations and their subsequent co-culture with A549 cells for functional characterization. (G) SEM images of sEVs derived from A549, A549CR, and CSCs. Scale bar: 200 nm. (H) NTA of sEVs from A549, A549CR, and CSCs showing size distribution. (I) Confocal microscopy images of A549 cells incubated with PKH67-labeled sEVs showing sEV uptake. Nuclei are stained with DAPI (blue), cytoskeletons with phalloidin (red), and sEVs with PKH67 (green). Scale bar: 20 µm. (J) Western blot analysis of stem cell markers in A549 cells after incubation with sEVs from different origins. (K) Flow cytometry analysis of CD44 and CD133 expression in A549 cells treated with sEVs. (L) Quantification of cells positive for CD44 and CD133 after treatment with sEVs from different sources. Data are presented as mean ± SD (ns: not significant, *p < 0.05, ***p < 0.001, n = 3).

To identify the primary contributors to tumor drug resistance, we compared the stemness properties of A549 cisplatin-resistant cells (A549CR) with their chemosensitive counterparts. A549CR cells exhibited significantly enhanced stemness traits, as shown by increased clonogenic survival and sphere formation compared to chemosensitive A549 cells (Figure S1A-C). We enriched the CSC population through sphere culture from A549 and H1299 tumor-bearing mice and verified the enrichment using western blotting and flow cytometry to detect stem cell transcription factors (NANOG, OCT4, SOX2) and CSC surface markers (CD44, CD133) (Figure 1A-C, Figure S1D-F).

Next, we evaluated the drug resistance profiles of these cell populations. Upon exposure to cisplatin and paclitaxel, CSCs exhibited marked resistance, retaining high cell viability and displaying minimal apoptotic activity compared to their chemosensitive counterparts (Figure S1G-J). The IC50 values for cisplatin were 4.3 µg/mL (A549), 17 µg/mL (A549CR), 60 µg/mL (A549-CSCs), 1.328 µg/mL (H1299), and 12.21 µg/mL (H1299-CSC). Similarly, the IC50 values for paclitaxel were 10.93 ng/mL, 15.93 ng/mL, 42.98 ng/mL, 53.04 ng/mL, and 3740 ng/mL, respectively (Figure 1D, Figure S1I-J).

To investigate the mechanisms underlying CSC resistance, we conducted comparative proteomic profiling of A549-sensitive cells and CSCs using liquid chromatography-mass spectrometry (Figure 1A). Of the 6,761 proteins identified and quantified, 1,003 were upregulated and 1,173 were downregulated in CSCs (Figure S1K). Gene Ontology (GO) enrichment analysis revealed upregulation of extracellular vesicle-related pathways in CSCs, suggesting a role for CSC-derived sEVs in stemness propagation (Figure 1E). To further explore this hypothesis, we isolated and characterized sEVs from A549, A549CR, and A549 CSC culture media (Figure 1F). Scanning electron microscopy confirmed the characteristic cup-shaped morphology of sEVs (Figure 1G). Nanoparticle tracking analysis and nanoflow cytometry demonstrated that sEVs consistently fell within the typical size range of 30-200 nm (Figure 1H and Figure S1L). Western blotting confirmed the enrichment of sEV markers (CD63, CD81) and the absence of contamination markers (GRP94, Calnexin), validating the purity of isolated sEVs (Figure S1M).

To assess the functional effects of sEVs, we labeled them with the green fluorescent dye PKH67 and observed their uptake by A549 cells (Figure 1I). Notably, cells treated with CSC-derived sEVs exhibited increased expression of stemness markers (OCT4, NANOG, SOX2) compared to those treated with sEVs from A549 or A549CR cells (Figure 1J). Flow cytometry confirmed higher expression of CD44 and CD133 and reduced apoptosis when exposed to chemotherapy in CSC-sEV-treated cells (Figure 1K-L, Figure S1N-O). Further analysis excluded the possibility that sEVs carried CD44 and CD133 directly, as no differences were detected in the protein levels within the sEVs themselves; however, significant variations were observed in the expression of these proteins in recipient cells treated with sEVs from different sources (Figure S1P).

**Figure 2:**
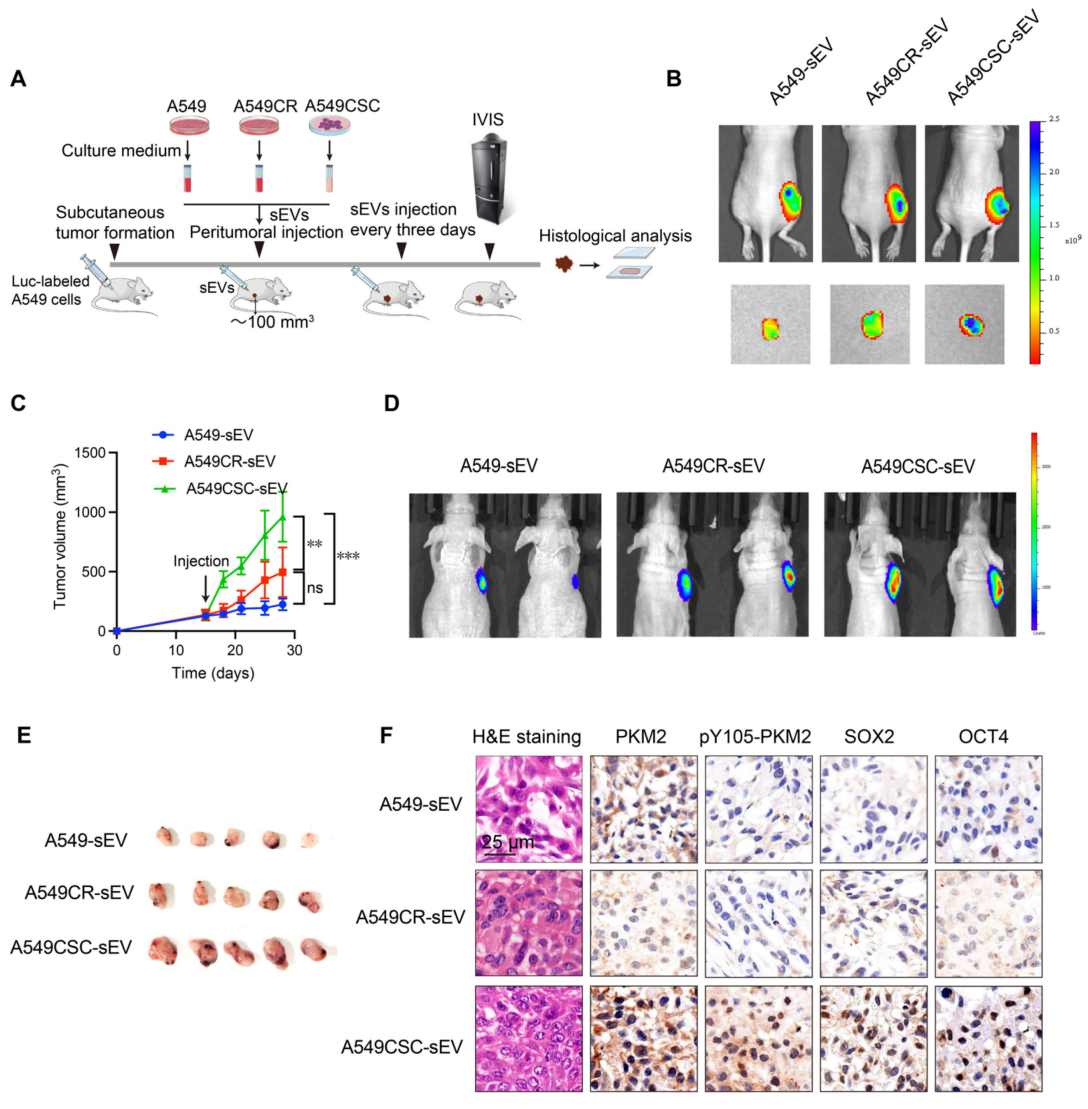
***In vivo* effects of sEVs on tumor growth and stemness.** (A) Schematic diagram of the *in vivo* experimental design. A549 cells were injected subcutaneously into nude mice to establish tumors. sEVs derived from A549, A549CR, and A549CSC were injected peritumorally every three days. Tumor growth was monitored using IVIS imaging, and histological analyses were performed post-harvest. (B) Representative IVIS imaging showing fluorescence signals in tumors treated with Dir-labeled sEVs from different origins. The images include *in vivo* and *ex vivo* fluorescence signals after sEV injection. (C) Tumor growth curves showing tumor volume changes over time in mice treated with sEVs from different sources. Data are presented as mean ± SD (ns: not significant, **p < 0.01, ***p < 0.001, n = 5). Comparisons between groups were analyzed using one-way ANOVA followed by Tukey's post-hoc test. (D) Representative IVIS images showing *in vivo* bioluminescent signal intensity in tumor-bearing mice treated with sEVs derived from A549, A549CR, or A549CSC. (E) Representative images of harvested tumors from each group after treatment with sEVs. (F) Histological and immunohistochemical analyses of tumors. H&E staining shows tumor morphology, while immunohistochemical staining shows PKM2, pY105-PKM2, SOX2, and OCT4. Scale bar: 25 µm.

Consistent with these *in vitro* results, *in vivo* experiments demonstrated that sEVs can propagate stemness in the tumor microenvironment. A549 cells implanted subcutaneously in nude mice were treated with sEVs from various cellular sources (Figure 2A). To confirm sEV uptake by tumors, we labeled sEVs with the lipophilic near-infrared dye Dir and detected fluorescence signals in tumor-bearing mice via IVIS imaging after two injections (Figure 2B). Tumors treated with CSC-derived sEVs exhibited accelerated growth, as demonstrated by tumor growth curves and confirmed via *in vivo* and *ex vivo* imaging (Figure 2C-E). Immunohistochemical analysis of harvested tumors revealed elevated levels of stemness markers (OCT4, SOX2) in CSC-sEV-treated groups (Figure 2F).

These findings collectively indicate that CSC-derived sEVs promote the dissemination of stemness traits, which replenish the CSC pool and may explain the persistence of stemness even after the original CSC population is depleted.

### Phosphorylation of PKM2 at Y105 is associated with stemness features and predicts poor clinical outcomes in NSCLC

**Figure 3:**
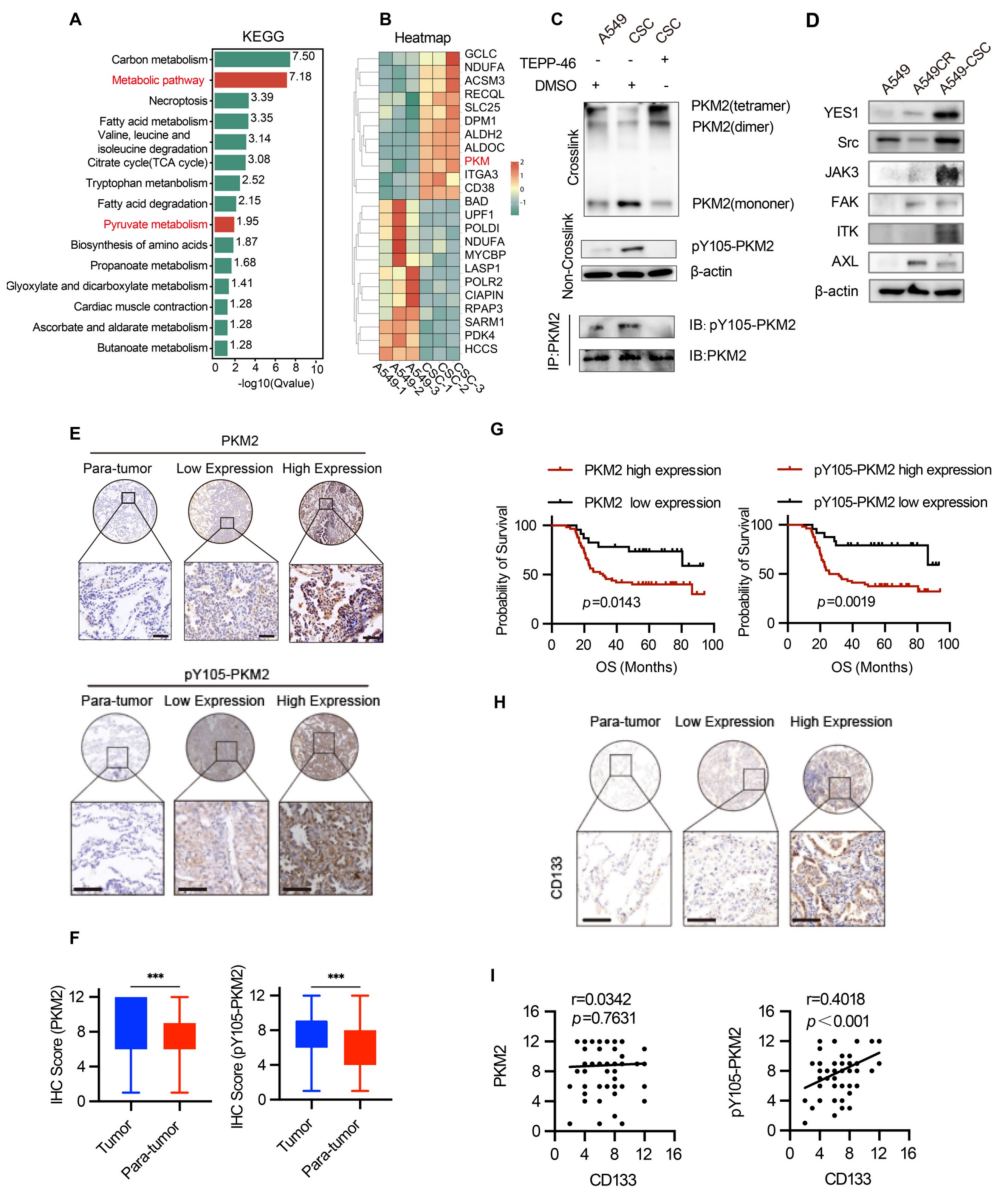
** Analysis of PKM2 and its phosphorylation in NSCLC cells and tissues.** (A) KEGG pathway enrichment analysis of differentially expressed proteins in A549 chemosensitive cells and CSCs. (B) Heatmap depicting proteomic analysis of A549 chemosensitive cells and CSCs. (C) Western blot analysis of PKM2 and pY105-PKM2 in A549, A549CR, and CSCs, with or without TEPP-46 treatment. Crosslinking experiments show PKM2 configurations, and immunoprecipitation (IP) indicates phosphorylation at Y105. (D) Western blot analysis of selected kinases (YES1, Src, JAK3, FAK, ITK, AXL) in A549, A549CR, and CSCs. (E) Representative immunohistochemical staining of PKM2 and pY105-PKM2 in tumor and peritumoral tissues. Scale bars: 50 µm. (F) Box plots showing IHC scores of PKM2 and pY105-PKM2 in tumor and peritumoral tissues. (G) Kaplan-Meier survival curves comparing overall survival (OS) of NSCLC patients based on PKM2 and pY105-PKM2 expression levels. (H) Representative immunohistochemistry staining of CD133 in tumor and peritumoral tissues. Scale bars: 50 µm. (I) Scatter plots showing the relationship between CD133 expression and PKM2 or pY105-PKM2 in tumor tissues.

Proteomic analysis of A549 chemosensitive cells and CSCs revealed significant enrichment of the pyruvate metabolic pathway and marked upregulation of PKM protein in CSCs (Figure 3A-B). Considering PKM2's pivotal role in cancer metabolism, we focused on its post-translational modifications, specifically phosphorylation at Y105, which influences its dimeric/monomeric state and oncogenic functions 29. Our results showed that PKM2 is upregulated in CSCs and predominantly exists in dimer/monomer forms following crosslinking in A549 and H1299 cells (Figure 3C and Figure S2A). Notably, phosphorylation of PKM2 at the Y105 site promotes its dimer/monomer configuration, a state associated with enhanced glycolytic activity and tumorigenic potential 30. Western blot analysis confirmed enhanced phosphorylation of PKM2 at Y105 in CSCs compared to chemosensitive cells (Figure 3C).

To further investigate the functional implications of PKM2 phosphorylation, we utilized TEPP-46, an allosteric activator that stabilizes PKM2 in its tetrameric form and inhibits its dimeric configuration 31. Treatment with TEPP-46 reduced the dimeric form of PKM2 while increasing pY105-PKM2 levels, suggesting a feedback mechanism that maintains PKM2 phosphorylation in CSCs (Figure 3C). PKM2 immunoprecipitation of the samples in Figure 3C confirmed the specificity of Y105 phosphorylation. After normalizing PKM2 levels, we observed a significant increase in Y105 phosphorylation in CSCs, which was attenuated by TEPP-46 treatment, further supporting the role of PKM2 phosphorylation in CSC biology (Figure 3C). In the animal experiments described earlier (Figure 2A), we found that treatment of A549 tumors with CSC-derived sEVs significantly increased PKM2 levels, particularly the phosphorylation of PKM2 at Y105, compared to tumors treated with sEVs from A549 or A549CR cells (Figure 2F). This suggests that CSC-derived sEVs carry regulatory factors that modulate PKM2 phosphorylation in recipient cells, thereby promoting stemness traits. We hypothesized that the elevated pY105-PKM2 levels in CSCs result from aberrant activation of oncogenic tyrosine kinases. Previous studies have shown that YES1, Src, JAK3, FAK, ITK, and AXL can induce PKM2 phosphorylation at Y105 28. Consistent with this, our western blot analysis revealed increased expression of these six proteins in CSCs compared to chemosensitive cells (Figure 3D). Similar results were observed in H1299 cells, indicating a conserved mechanism across different NSCLC models (Figure S2B).

To validate our findings clinically, we analyzed tumor and peritumoral tissue samples from 80 NSCLC patients. Tissue microarrays were constructed, and immunohistochemical staining was performed for PKM2, pY105-PKM2, and the stemness marker CD133. Statistical analysis revealed that tumor tissues exhibited significantly higher levels of PKM2 and pY105-PKM2 than adjacent non-tumorous tissues (Figure 3E-F). Elevated pY105-PKM2 levels were strongly associated with lymph node metastasis, chemotherapy recurrence, and advanced TNM stage (Table S1 and Table S2). Notably, pY105-PKM2 emerged as a robust predictor of reduced overall survival, displaying a stronger correlation with poor overall survival outcomes than total PKM2 (*p* = 0.0019 and *p* = 0.0143, respectively) (Figure 3G). Importantly, only pY105-PKM2 expression, not total PKM2, was associated with the presence of the stemness marker CD133 (Figure 3H-I). These findings highlight the critical role of pY105-PKM2 in promoting stemness traits and influencing NSCLC patient prognosis.

In summary, these results demonstrate that PKM2 phosphorylation at Y105 is a key regulator of stemness in NSCLC. The elevated levels of pY105-PKM2 in CSCs, its association with stemness markers, and its strong correlation with poor clinical outcomes underscore its potential as a therapeutic target and prognostic biomarker.

### Phosphorylated PKM2 facilitates the acquisition of CSC-like phenotypes in NSCLC cells *in vitro* and *in vivo*

**Figure 4:**
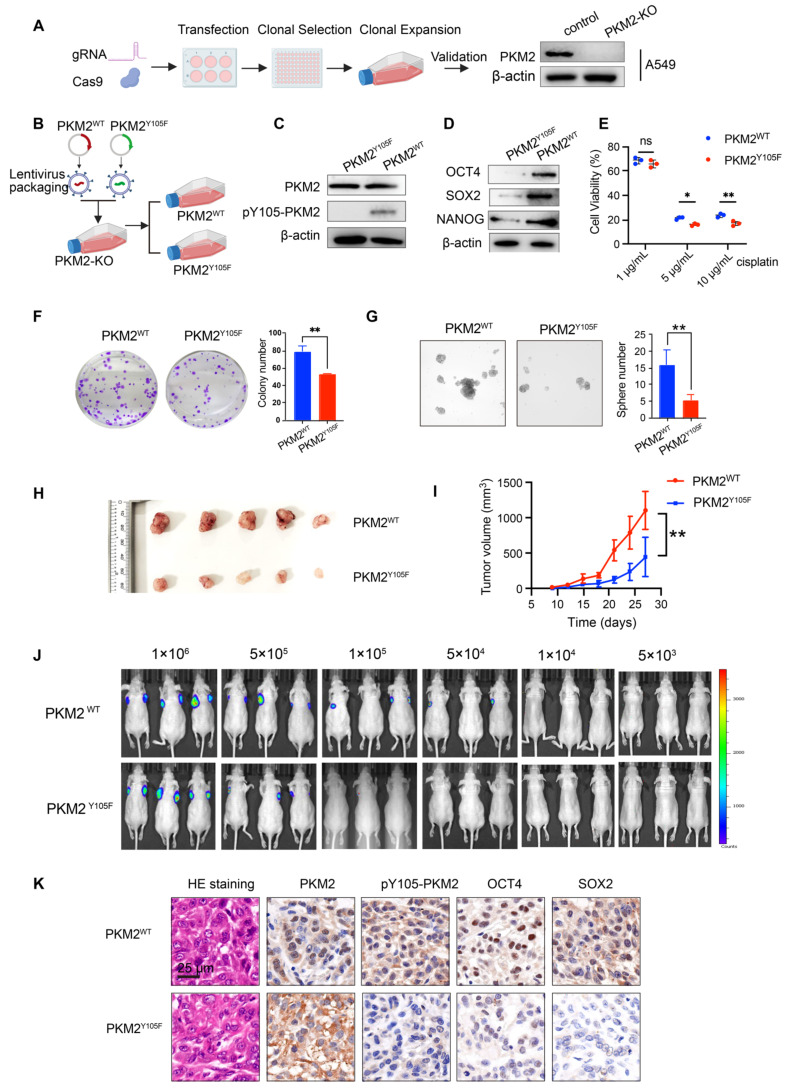
** Generation and characterization of PKM2^WT^ and PKM2^Y105F^ cell lines and their impact on NSCLC stemness and tumorigenicity.** (A) Schematic of CRISPR-Cas9-based PKM2 knockout in A549 cells followed by lentiviral transduction of PKM2^WT^ or PKM2^Y105F^ plasmids and western blot validation of PKM2 knockout.(B) Workflow of lentiviral packaging, transduction, and selection of PKM2^WT^ and PKM2^Y105F^ stable cell lines. (C) Western blot analysis showing expression of PKM2 and pY105-PKM2 in PKM2^WT^ and PKM2^Y105F^ cell lines. (D) Western blot analysis of stemness markers (OCT4, SOX2, and NANOG) in PKM2^WT^ and PKM2^Y105F^ cell lines. (E) Cell viability assays of PKM2^WT^ and PKM2^Y105F^ cells following cisplatin treatment. Data are presented as mean ± SD (ns: not significant, *p < 0.05, ** *p* < 0.01, n = 3). (F) Colony formation assay of PKM2^WT^ and PKM2^Y105F^ cells. Representative images and quantified colony numbers are shown. Data are presented as mean ± SD (**p < 0.01, n = 3). (G) Sphere formation assay of PKM2^WT^ and PKM2^Y105F^ cells. Representative images and quantified sphere numbers are shown. Data are presented as mean ± SD (**p < 0.01, n = 3). (H) Representative images of harvested subcutaneous xenograft tumors derived from PKM2^WT^ and PKM2^Y105F^ cells. (I) Tumor growth curves showing tumor volume over time for PKM2^WT^ and PKM2^Y105F^ xenografts. Data are presented as mean ± SD (**p < 0.01, n = 6). (J) Limiting dilution assay using bioluminescence imaging of subcutaneous xenografts with decreasing cell numbers (1×10⁶ to 5×10³) of PKM2^WT^ and PKM2^Y105F^ cells. (K) Immunohistochemical analysis of xenograft tumors showing H&E staining, PKM2, pY105-PKM2, and stemness markers (OCT4, SOX2). Scale bar = 25 μm.

To determine whether pY105-PKM2 drives stemness in lung cancer cells, we genetically modified A549 and H1299 cells to stably overexpress either wild-type PKM2 (PKM2^WT^) or its non-phosphorylatable mutant, PKM2^Y105F^, where tyrosine at position 105 was replaced with phenylalanine. First, endogenous PKM2 was ablated using CRISPR-Cas9 gene editing, as shown in Figure 4A and Figure S3A. Subsequently, plasmids encoding PKM2^WT^ or PKM2^Y105F^ were introduced into PKM2-knockout cells via lentiviral transduction (Figure 4B). Stable cell lines overexpressing PKM2^WT^ or PKM2^Y105F^ were established through puromycin selection and validated by western blotting (Figure 4C and Figure S3B).

To assess the impact of PKM2 phosphorylation status on chemoresistance, we performed cell viability assays following cisplatin treatment. Cells expressing PKM2^WT^ exhibited significantly higher drug resistance than those expressing PKM2^Y105F^ (Figure 4E and Figure S3C). Stemness properties were evaluated using colony formation assay, spheroid formation assay, and western blotting for stemness markers (OCT4, SOX2, and NANOG) (Figure 4D-G and Figure S3D-F). Cells expressing PKM2^Y105F^ showed a marked reduction in stemness traits compared to PKM2^WT^-expressing cells, indicating that phosphorylation at Y105 is critical for maintaining stemness.

We used subcutaneous xenograft models for* in vivo* validation. PKM2^WT^-expressing cells demonstrated enhanced tumorigenicity, as evidenced by increased tumor volume (Figure 4H-I). To further assess stemness *in vivo*, we conducted a limiting dilution assay—the gold standard for stemness evaluation—by subcutaneously inoculating varying numbers of cells and assessed tumor formation after three weeks. *In vivo* bioluminescence imaging and statistical analysis revealed that 1×10⁵ and 5×10⁴ PKM2^Y105F^ cells exhibited a significantly reduced ability to initiate tumors compared to PKM2^WT^ cells (Figure 4J and Figure S3G). Immunohistochemical analysis of xenograft tumors showed elevated expression of OCT4 and SOX2 in tumors derived from PKM2^WT^-expressing cells, further supporting the role of pY105-PKM2 in promoting stemness (Figure 4K).

Collectively, these findings show that pY105-PKM2 is a critical regulator of stem-like properties in NSCLC, driving increased expression of stemness markers, enhanced tumorigenicity, and chemoresistance *in vitro* and *in vivo*.

### sEV-mediated pY105-PKM2 induces slow cell cycle, metabolic remodeling, and promotes chemoresistance and stemness in NSCLC

Our previous studies demonstrated that drug resistance can be transferred via sEVs carrying PKM2 from drug-resistant cells to chemosensitive cells 27. Building on these findings, we hypothesized that the phosphorylated form of PKM2 at tyrosine 105 (pY105-PKM2), a known driver of tumor stemness, could contribute to the transmission of stem-like traits via sEVs, thereby replenishing the CSC pool. To test this hypothesis, we isolated sEVs from A549 cells overexpressing either wild-type PKM2 (A549-PKM2^WT^), the Y105F mutant variant (A549-PKM2^Y105F^), or control cells. These sEVs were incubated with chemosensitive A549 cells for 48 h (Figure S3H). Flow cytometry revealed increased CD44 and CD133 expression in cells treated with A549-PKM2^WT^ sEVs (Figure S3I-J). These results indicate that PKM2^WT^-derived sEVs promote the acquisition of stem-like properties in recipient cells.

To assess the influence of sEVs on chemoresistance, we incubated A549 cells with sEVs derived from A549-PKM2^WT^, A549-PKM2^Y105F^, or control cells for 48 h, followed by treatment with cisplatin or paclitaxel for an additional 24 h (Figure S3K). Cell viability assays demonstrated that cells treated with A549-PKM2^WT^-derived sEVs displayed significantly higher survival rates than those treated with sEVs from A549-PKM2^Y105F^ or control cells, indicating enhanced chemoresistance (Figure S3K). Consistent with these findings, flow cytometry analysis showed that apoptosis levels were significantly reduced in A549 cells exposed to A549-PKM2^WT^-derived sEVs compared to those treated with A549-PKM2^Y105F^ or control sEVs (Figure S3L-M).

Collectively, these results highlight the role of sEV-mediated delivery of pY105-PKM2 in promoting chemotherapeutic resilience in recipient cells.

**Figure 5:**
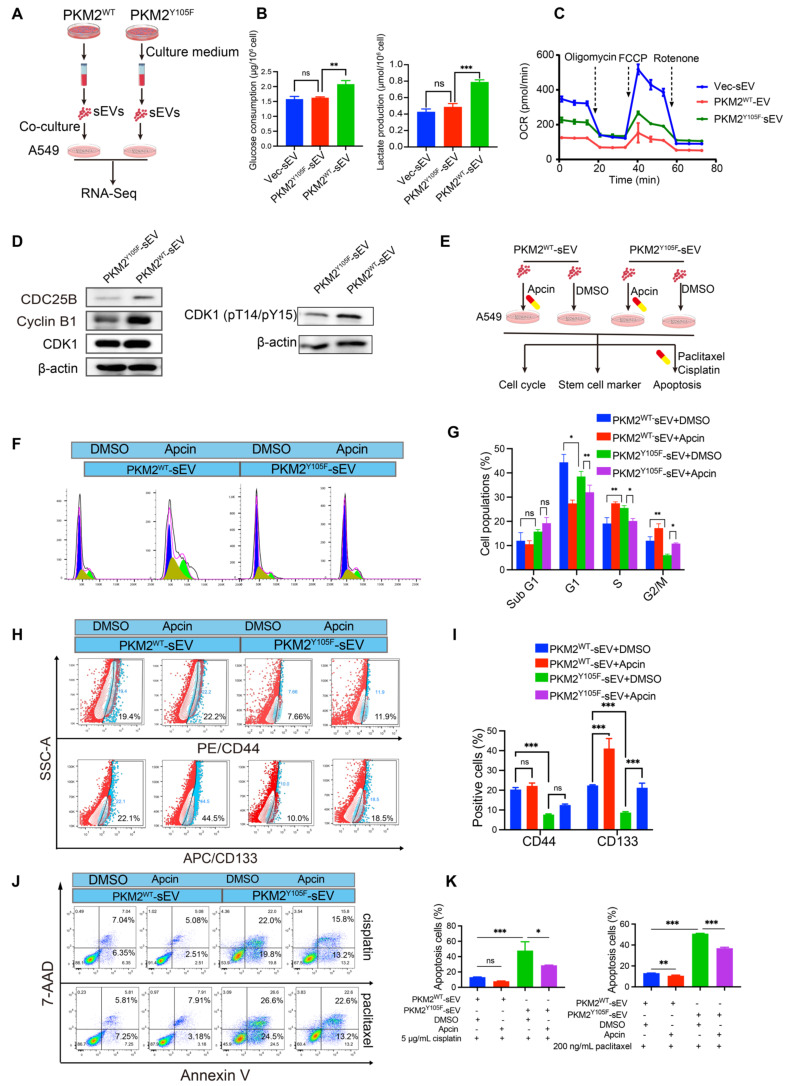
** sEV-mediated pY105-PKM2 induces slow cell cycle, metabolic remodeling, and promotes chemoresistance and stemness in NSCLC.** (A) Schematic representation of the experimental setup. A549 cells were treated with sEVs derived from A549-PKM2^WT^ and A549-PKM2^Y105F^ cells, followed by transcriptomic sequencing. (B) Glucose consumption and lactate production in A549 cells treated with Vec-sEV, PKM2^WT-sEV, or PKM2^Y105F-sEV. Data are presented as mean ± SD (ns, not significant, **p < 0.01, ***p < 0.001, n = 3). (C) OCR of A549 cells treated with Vec-sEV, PKM2^WT^-sEV, or PKM2^Y105F^-sEV under sequential injections of oligomycin, FCCP, and rotenone. (D) Western blot analysis of Cyclin B1, CDC25B, total CDK1, and CDK1 phosphorylated at Thr14 and Tyr15 in A549 cells treated with PKM2^WT^-sEV or PKM2^Y105F^-sEV. (E) Schematic representation of Apcin treatment experiments designed to assess the role of APC/CDC20 in sEV-induced cell cycle regulation, stemness, and chemoresistance. (F) Flow cytometry analysis of cell cycle distribution in A549 cells treated with PKM2^WT^-sEV or PKM2^Y105F^-sEV, with or without Apcin treatment. (G) Quantification of cell populations in different phases of the cell cycle (Sub G1, G1, S, G2/M) based on flow cytometry analysis. Data are presented as mean ± SD (ns, not significant, *p < 0.05, **p < 0.01, n = 3). (H) Flow cytometry analysis of CD44 and CD133 expression in A549 cells treated with PKM2^WT^-sEV or PKM2^Y105F^-sEV, with or without Apcin treatment. (I) Quantification of CD44- and CD133-positive cells in A549 cells treated as described in Panel H. Data are presented as mean ± SD (ns, not significant, ***p < 0.001, n = 3). (J) Flow cytometry analysis of apoptotic cells in A549 cells treated with sEVs, followed by cisplatin or paclitaxel treatment, with or without Apcin. (K) Quantification of apoptotic cells in A549 cells treated as described in Panel J. Data are presented as mean ± SD (ns, not significant, *p < 0.05, **p < 0.01, ***p < 0.001, n = 3).

To investigate the molecular mechanisms underlying these effects, we performed transcriptomic sequencing on A549 cells treated with sEVs from A549-PKM2^WT^ and A549-PKM2^Y105F^ (Figure 5A). Of the 629 genes differentially expressed between the two treatment groups, 196 genes were upregulated and 433 were downregulated in the A549-PKM2^WT^ sEV group (Figure S4A-B; fold-change ≥ 2, adjusted *p* ≤ 0.01). A heatmap of representative genes revealed alterations in biological processes critical for cell cycle regulation, vesicular transport, protein biosynthesis, and post-translational modifications (Figure S4C). GO and KEGG pathway analyses highlighted significant enrichment in cellular metabolic processes, protein metabolic processes, pyruvate metabolism, and cell cycle regulation (Figure S4D-E). Gene Set Enrichment Analysis (GSEA) further demonstrated a positive enrichment of pyruvate metabolism in the A549-PKM2^WT^ sEV treatment group, suggesting enhanced metabolic activity in this condition. Conversely, cell cycle-related pathways, including G1/S phase transition and cell cycle checkpoint, showed a negative enrichment in the A549-PKM2^Y105F^ sEV treatment group, indicating disrupted cell cycle progression (Figure S4F). Reactome pathway analysis reinforced these findings, showing that pathways associated with APC/C-CDC20-mediated regulation of the cell cycle were prominently altered (Figure S5G).

To investigate whether RNA enclosed in sEVs contributes to cell cycle regulation in recipient cells, we performed miRNA sequencing on sEVs derived from A549-PKM2^WT^ and A549-PKM2^Y105F^ cells, given that miRNAs are the primary regulatory RNAs in EVs. A total of 62 sEV-associated miRNAs were identified with significant differences (Figure S5A-B). KEGG (Figure S5C) and Reactome (Figure S5D) analyses of their target genes revealed enrichment in GPCR signaling and SUMOylation regulation rather than direct involvement in cell cycle control. Therefore, we propose that sEVs mediate APC/C-dependent cell cycle regulation in recipient cells predominantly through the delivery of Y105-phosphorylated PKM2.

A549-PKM2^WT^ sEV treatment enhanced glycolysis, as demonstrated by increased glucose consumption and lactate production accompanied by weakened mitochondrial oxidative phosphorylation (Figure 5B-C). Inhibition of APC/CDC20 activity is known to impede the degradation of its substrates, delay the mitosis-to-G1 phase transition, and thereby induce slower cell cycle progression, ultimately contributing to chemotherapeutic resistance 32-34. Western blot analysis following A549-PKM2^WT^ sEV treatment showed upregulated Cyclin B1, a known substrate of APC/CDC20 34, suggesting inhibition of APC/CDC20 activity (Figure 5D). Although total CDK1 levels remained unchanged, an increase in CDK1 Thr14 and Tyr15 phosphorylation was observed in A549 cells treated with A549-PKM2^WT^ sEVs, with the upregulation of CDC25B further supporting CDK1 activity (Figure 5D). CDC25A/B-mediated dephosphorylation of CDK1 at Thr14 and Tyr15 is critical for CDK1 activation and the initiation of mitosis 35. Notably, flow cytometry analysis revealed that A549-PKM2^WT^ sEV treatment induced a slower cell cycle progression, characterized by a reduction in the proportion of mitotically active cells (G2/M phase), compared to cells treated with A549-PKM2^Y105F^ sEVs (Figure 5E-G).

These findings collectively indicate that the uptake of A549-PKM2^WT^-derived sEVs by recipient cells modulates the expression of cell cycle proteins at the transcriptional level, driving these cells into a slower cell cycle phase.

To determine whether the slow cell cycle induced by A549-PKM2^WT^ sEVs contributes to enhanced resistance and stemness in recipient cells, we treated chemosensitive A549 cells with sEVs from different sources, followed by Apcin, a small-molecule inhibitor of APC/CDC20 activity (Figure 5E). Apcin treatment resulted in a prolonged G2/M phase in cells treated with A549-PKM2^Y105F^-derived sEVs, leading to slower cell cycle progression (Figure 5F-G) and enhanced stemness and chemoresistance in recipient cells (Figure 5H-K) - effects similar to those observed with A549-PKM2^WT^ sEV treatment. These results suggest that inhibiting the APC/CDC20 pathway by A549-PKM2^WT^ sEVs drives enhanced chemoresistance and stemness in recipient cells.

**Figure 6:**
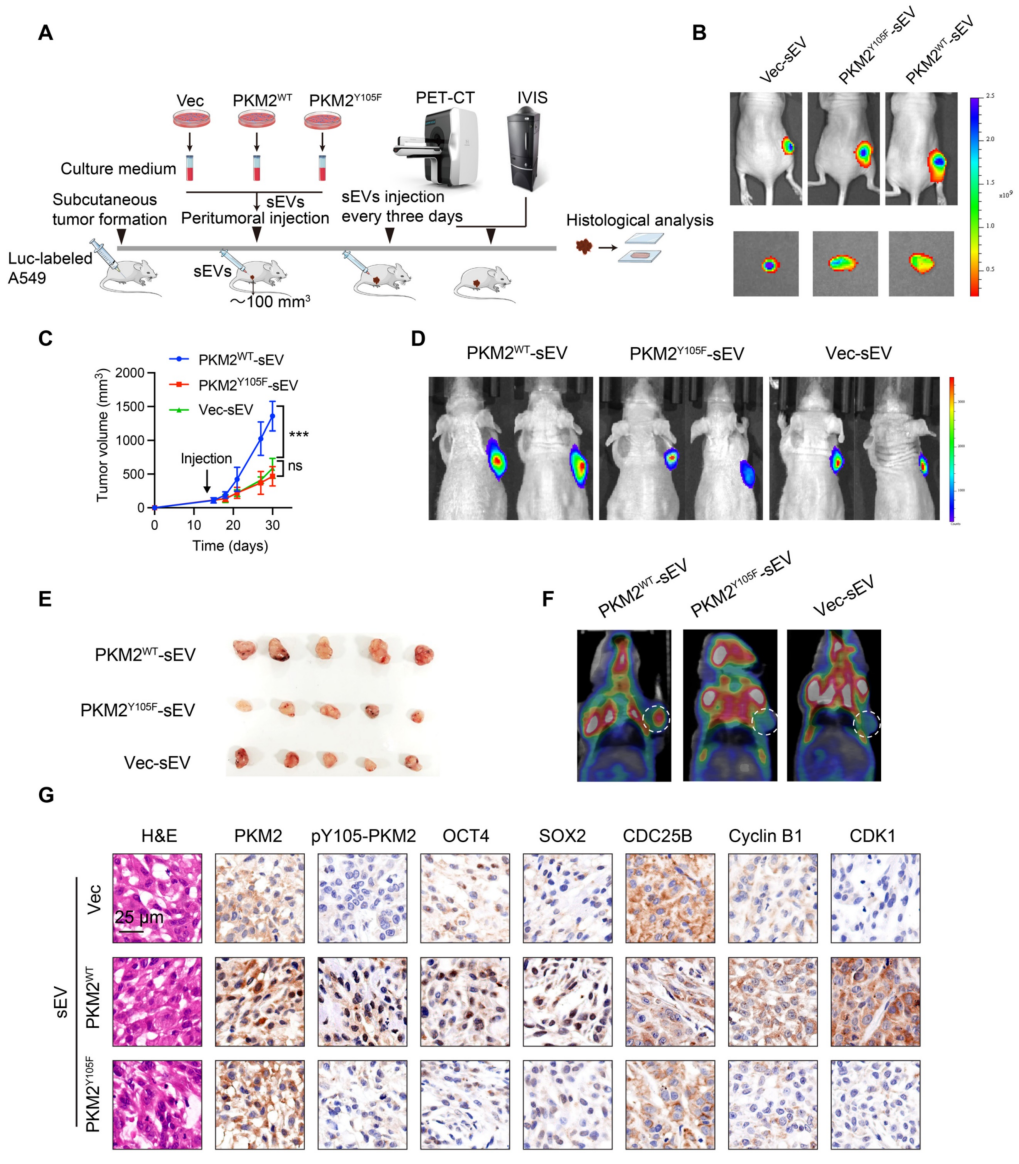
***In vivo* assessment of the effects of sEVs derived from PKM2^WT^ and PKM2^Y105F^ cells on tumor growth, glycolytic activity, and stemness.** (A) Schematic representation of the *in vivo* experimental design. Chemosensitive A549 cells were subcutaneously implanted into nude mice, and upon tumor volumes reaching ~100 mm³, sEVs derived from Vec, PKM2^WT^, or PKM2^Y105F^ cells were administered peritumorally every three days. Tumor growth was monitored, followed by ¹⁸F-FDG PET-CT and IVIS imaging and histological analysis of harvested tumors. (B) Representative IVIS imaging showing fluorescence signals in tumors treated with Dir-labeled sEVs from different origins. The images include *in vivo* and *ex vivo* fluorescence signals after sEV injection. (C) Tumor growth curves depicting tumor volumes over time in mice treated with different sEVs. Data are presented as mean ± SD (ns, not significant, ***p < 0.001, n = 5). (D) Representative IVIS images showing bioluminescent signals from subcutaneous tumors at the experimental endpoint in mice treated with Vec-sEV, PKM2^WT^-sEV, or PKM2^Y105F^-sEV. (E) Images of harvested tumors from mice treated with different sEVs. (F) Representative ¹⁸F-FDG PET-CT scans showing glycolytic activity in tumors treated with different sEVs. (G) Histological analysis of tumor tissues, including hematoxylin and eosin (HE) staining and immunohistochemistry for PKM2, pY105-PKM2, stemness markers OCT4 and SOX2, and cell cycle regulators CDC25B, Cyclin B1, and CDK1. Scale bar: 25 µm.

In complementary *in vivo* experiments, we subcutaneously implanted chemosensitive A549 cells into nude mice and administered sEVs from different sources when tumors reached ~100 mm³ (Figure 6A). Tumors treated with A549-PKM2^WT^-derived sEVs exhibited enhanced stemness, as evidenced by increased tumor volume compared to tumors treated with A549-PKM2^Y105F^ sEVs (Figure 6B—E). Additionally, ¹⁸F-FDG PET-CT scans revealed elevated glycolytic metabolism in tumors treated with A549-PKM2^WT^ sEVs (Figure 6F). Immunohistochemistry analysis further demonstrated increased expression of the stemness markers OCT4 and SOX2 along with the accumulation of Cyclin B1, CDC25B, and CDK1 (Figure 6G).

Taken together, these results indicate that A549-PKM2^WT^-derived sEVs promote metabolic reprogramming and modulate the expression of cell cycle-related genes in recipient tumor cells, facilitating their survival under chemotherapeutic stress and expanding the stem cell pool.

### IQGAP1 serves as an adaptor which bridges phosphorylated PKM2 to TSG101

**Figure 7:**
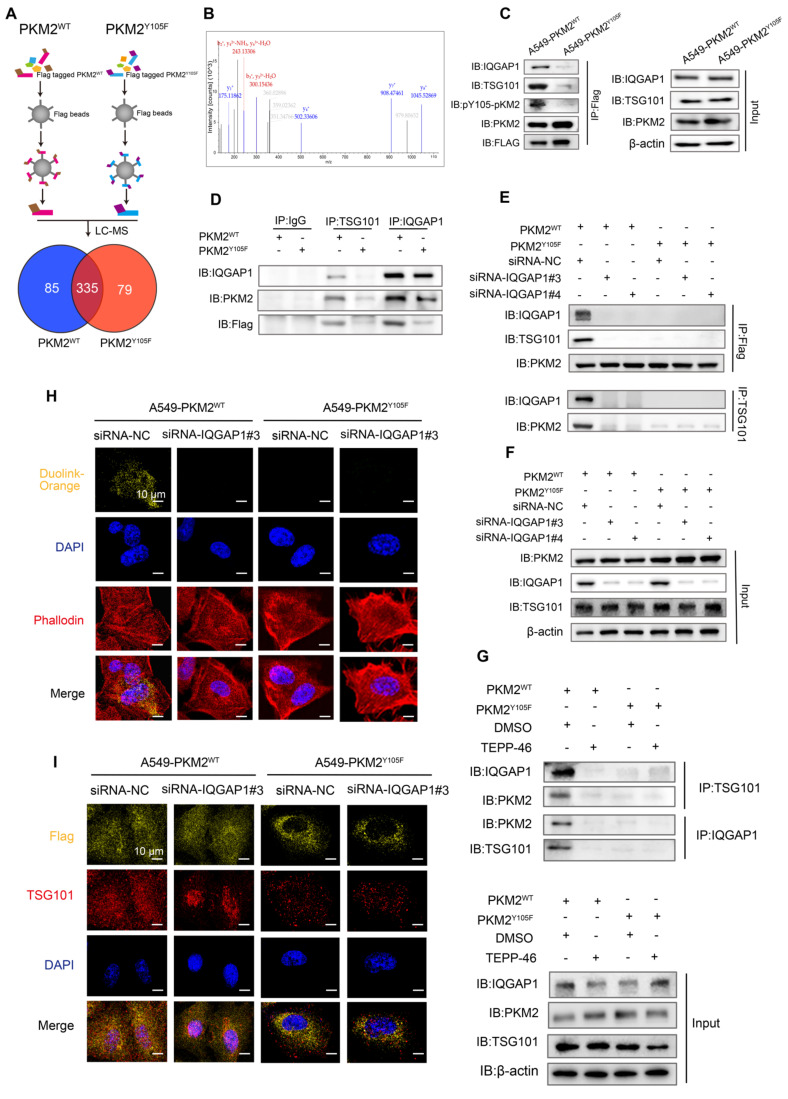
** IQGAP1 links phosphorylated PKM2 to TSG101.** (A) Experimental workflow for immunoprecipitation and mass spectrometry analysis. Flag-tagged PKM2^WT^ and PKM2^Y105F^ proteins were immunoprecipitated using anti-Flag magnetic beads, followed by liquid chromatography-mass spectrometry. The Venn diagram illustrates the overlap and unique binding partners of PKM2^WT^ and PKM2^Y105F^. (B) Mass spectrometry results highlighting the binding of IQGAP1 exclusively to PKM2^WT^. (C) Co-immunoprecipitation of IQGAP1, pY105-PKM2, and TSG101 in A549-PKM2^WT^ and A549- PKM2^Y105F^ cells. Total protein input is shown for comparison. (D) Immunoprecipitation analysis of interactions between IQGAP1, TSG101, and PKM2 in A549- PKM2^WT^ and A549- PKM2^Y105F^ cells. (E) Co-immunoprecipitation results following siRNA-mediated knockdown of IQGAP1 in A549-PKM2^WT^ and A549-PKM2^Y105F^ cells. (F) Total protein input levels of IQGAP1, TSG101, and PKM2 in A549- PKM2^WT^ and A549- PKM2^Y105F^ cells, with or without IQGAP1 knockdown. (G) Immunoprecipitation analysis showing the effect of TEPP-46 treatment on interactions between IQGAP1, PKM2, and TSG101 in A549- PKM2^WT^ and A549- PKM2^Y105F^ cells. (H) DuoLink proximity ligation assay detecting interactions between PKM2 and TSG101. Signals (yellow) are shown in A549- PKM2^WT^ and A549- PKM2^Y105F^ cells, with and without IQGAP1 knockdown. DAPI (blue) marks nuclei, and phalloidin (red) labels actin filaments. Scale bar: 10 µm. (I) Immunofluorescence analysis showing colocalization of Flag-tagged PKM2^WT^ and PKM2^Y105F^ with TSG101 in A549 cells, with and without IQGAP1 knockdown. Scale bar: 10 µm.

We further investigated the mechanisms underlying the selective incorporation of pY105-PKM2 into sEVs. Flag-PKM2^WT^ and Flag-PKM2^Y105F^ proteins and their binding partners were immunoprecipitated using Anti-Flag beads. LC-MS/MS analysis of these complexes identified 335 proteins interacting with PKM2^WT^ and PKM2^Y105F^, of which 79 were exclusive to PKM2^Y105F^ and 85 to PKM2^WT^ (Figure 7A). Secondary mass spectrometry revealed that IQGAP1 binds exclusively to the phosphorylatable form of PKM2 (Figure 7B).

Previous research has shown that IQGAP1 mediates ESCRT-dependent cargo activation and loading of proteins, such as TSG101, into sEVs 36. We hypothesized that IQGAP1 serves as an adaptor linking pY105-PKM2 to TSG101. Immunoprecipitation assays confirmed that IQGAP1, pY105-PKM2, and TSG101 were pulled down together in A549-PKM2^WT^ cells but not in A549-PKM2^Y105F^ cells with mutated tyrosine 105 (Figure 7C and Figure S6A-B). IQGAP1 also co-immunoprecipitated with pY105-PKM2 and TSG101 in A549-PKM2^WT^ cells but not in A549-PKM2^Y105F^ cells (Figure 7D). Additionally, TSG101 pulled down IQGAP1 and pY105-PKM2 only in PKM2^WT^ cells (Figure 7D).

Gene silencing experiments with siRNA targeting IQGAP1 (siRNA-IQGAP1#3 and siRNA-IQGAP1#4) further revealed that the interaction between PKM2^WT^ and TSG101 was disrupted upon IQGAP1 knockdown (Figure S6C, Figure 7E-F). However, this knockdown had no effect on PKM2^Y105F^-TSG101 binding (Figure 7E-F). Similarly, TEPP-46 treatment, which stabilizes PKM2 in its tetrameric form and inhibits Y105 phosphorylation, disrupted the interaction between PKM2^WT^, IQGAP1, and TSG101, but not between PKM2^Y105F^ and TSG101 (Figure S6D and 7G).

A DuoLink proximity ligation assay confirmed that PKM2-TSG101 binding was exclusive to PKM2^WT^ cells and absent in PKM2^Y105F^ cells. This binding was abolished by IQGAP1 silencing, reinforcing the role of IQGAP1 as an adaptor linking pY105-PKM2 to TSG101 (Figure 7H). Immunofluorescence colocalization further supported these findings, showing colocalization of Flag-tagged proteins with TSG101 in A549-PKM2^WT^ cells, which was disrupted upon IQGAP1 knockdown (Figure 7I).

### IQGAP1 mediates the selective sorting of pY105-PKM2 into sEVs and promotes drug resistance in recipient cells

**Figure 8:**
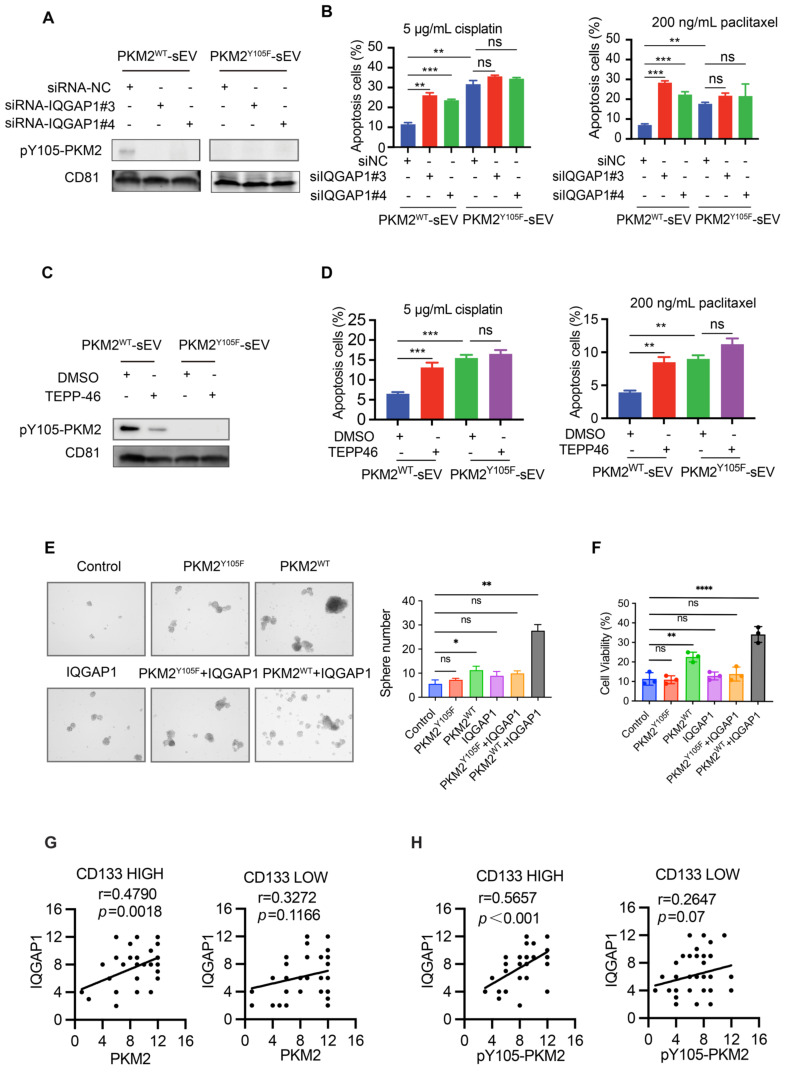
** IQGAP1 mediates the sorting of pY105-PKM2 into sEVs and synergistically promotes stemness and drug resistance.** (A) Western blot analysis of pY105-PKM2 in sEVs derived from A549-PKM2^WT^ or A549-PKM2^Y105F^ cells, with or without IQGAP1 silencing using siRNA (siRNA-NC, siRNA-IQGAP1#3, or siRNA-IQGAP1#4). (B) Flow cytometry analysis of apoptosis in recipient A549 cells treated with sEVs from IQGAP1-silenced or control A549-PKM2^WT^ cells, followed by treatment with cisplatin (5 µg/mL) or paclitaxel (200 ng/mL). (C) Western blot analysis of pY105-PKM2 in sEVs derived from A549-PKM2^WT^ or A549-PKM2^Y105F^ cells, with or without TEPP-46 treatment, which inhibits PKM2 phosphorylation at Y105. (D) Flow cytometry analysis of apoptosis in recipient A549 cells treated with sEVs from TEPP-46-treated A549-PKM2^WT^ cells, followed by cisplatin or paclitaxel treatment. (E) Sphere formation assay of A549 cells overexpressing IQGAP1, PKM2^WT^, PKM2^Y105F^, or combinations thereof. Representative images and quantification of sphere numbers are shown. (F) Cell viability assay of A549 cells overexpressing IQGAP1, PKM2^WT^, PKM2^Y105F^, or their combinations, following cisplatin treatment (5 µg/mL). (G) Correlation between IQGAP1 and PKM2 expression in CD133-high and CD133-low tumor samples. (H) Correlation between IQGAP1 and pY105-PKM2 expression in CD133-high and CD133-low tumor samples. Data (B-F) are presented as mean ± SD (n = 3). Statistical significance: ns, not significant; *p < 0.05, **p < 0.01, ***p < 0.001, ****p < 0.0001.

Given the potential interaction between IQGAP1 and pY105-PKM2, we investigated whether IQGAP1 regulates the incorporation of pY105-PKM2 into sEVs. Western blot analysis demonstrated that pY105-PKM2 was present in sEVs derived from A549-PKM2^WT^ but not A549-PKM2^Y105F^ cells (Figure 8A). siRNA-silencing of IQGAP1 significantly reduced the levels of pY105-PKM2 in sEVs (Figure 8A), suggesting that IQGAP1 is crucial for the selective sorting of pY105-PKM2 into sEVs.

To assess the functional consequences of IQGAP1 silencing for sEVs, we analyzed apoptosis in recipient A549 cells treated with sEVs derived from IQGAP1-silenced A549-PKM2^WT^ cells. These sEVs failed to enhance drug resistance, as evidenced by increased apoptosis rates upon cisplatin or paclitaxel treatment, consistent with reduced pY105-PKM2 levels in the recipient cells (Figure 8B, Figure S6E-F). Similarly, TEPP-46 treatment, which inhibits PKM2 phosphorylation at Y105, resulted in the loss of pY105-PKM2 in sEVs and rendered these sEVs ineffective in promoting drug resistance (Figure 8C-D, Figure S6G-H).

Collectively, these results establish that IQGAP1 mediates the selective sorting of pY105-PKM2 into sEVs, highlighting its critical role in modulating the functional properties of these vesicles during drug resistance.

### IQGAP1 and phosphorylated PKM2 synergistically promote stemness and malignant progression

Building on our findings that IQGAP1 mediates the selective incorporation of pY105-PKM2 into sEVs and enhances drug resistance in recipient cells, we investigated whether IQGAP1 and pY105-PKM2 act synergistically to promote stemness and malignant progression in NSCLC. We established cell lines overexpressing IQGAP1, as well as cell lines co-overexpressing IQGAP1 with either wild-type PKM2 (PKM2^WT^) or the Y105F mutant PKM2 (PKM2^Y105F^). Sphere formation assays revealed that IQGAP1 and PKM2^WT^, but not PKM2^Y105F^, synergistically enhanced stemness (Figure 8E). Chemoresistance assays further corroborated these findings, indicating that co-overexpression of IQGAP1 and PKM2^WT^ significantly promoted drug resistance compared to individual overexpression of either protein (Figure S7A, Figure 8F).

To assess the clinical significance of IQGAP1, we conducted immunohistochemical staining on 80 pairs of tumor and para-tumor tissue samples. Tumors were categorized into low and high IQGAP1 expression groups. We observed a significant upregulation of IQGAP1 in tumor tissues compared to adjacent para-tumor tissues (Figure S7B-C). Importantly, higher IQGAP1 expression levels were strongly associated with poor overall survival (*p* < 0.001, Figure S7D). Detailed clinicopathological analysis revealed that IQGAP1 expression was significantly associated with advanced TNM stage (*p* < 0.001), metastasis (*p* = 0.02), and recurrence (*p* < 0.001), suggesting a role in disease progression (Table S3). We further examined the interaction between IQGAP1 and PKM2 in CD133-high and CD133-low groups to explore their relationship in cancer stemness. IQGAP1 showed a significant positive correlation with PKM2 expression in the CD133-high group (*r* = 0.4790, *p* = 0.0018) but not in the CD133-low group (*r* = 0.3272, *p* = 0.1166) (Figure 8G). Notably, phosphorylated PKM2 (pY105-PKM2) was strongly correlated with IQGAP1 in the CD133-high group (*r* = 0.5657, *p* < 0.001) but not in the CD133-low group (Figure 8H).

These findings highlight the synergistic role of IQGAP1 and phosphorylated PKM2 in promoting stemness and driving malignant progression in NSCLC. The elevated expression of IQGAP1, particularly in conjunction with pY105-PKM2, underscores their importance in NSCLC pathogenesis and positions them as promising therapeutic targets to mitigate stemness and malignant progression.

## Discussion

**Figure 9:**
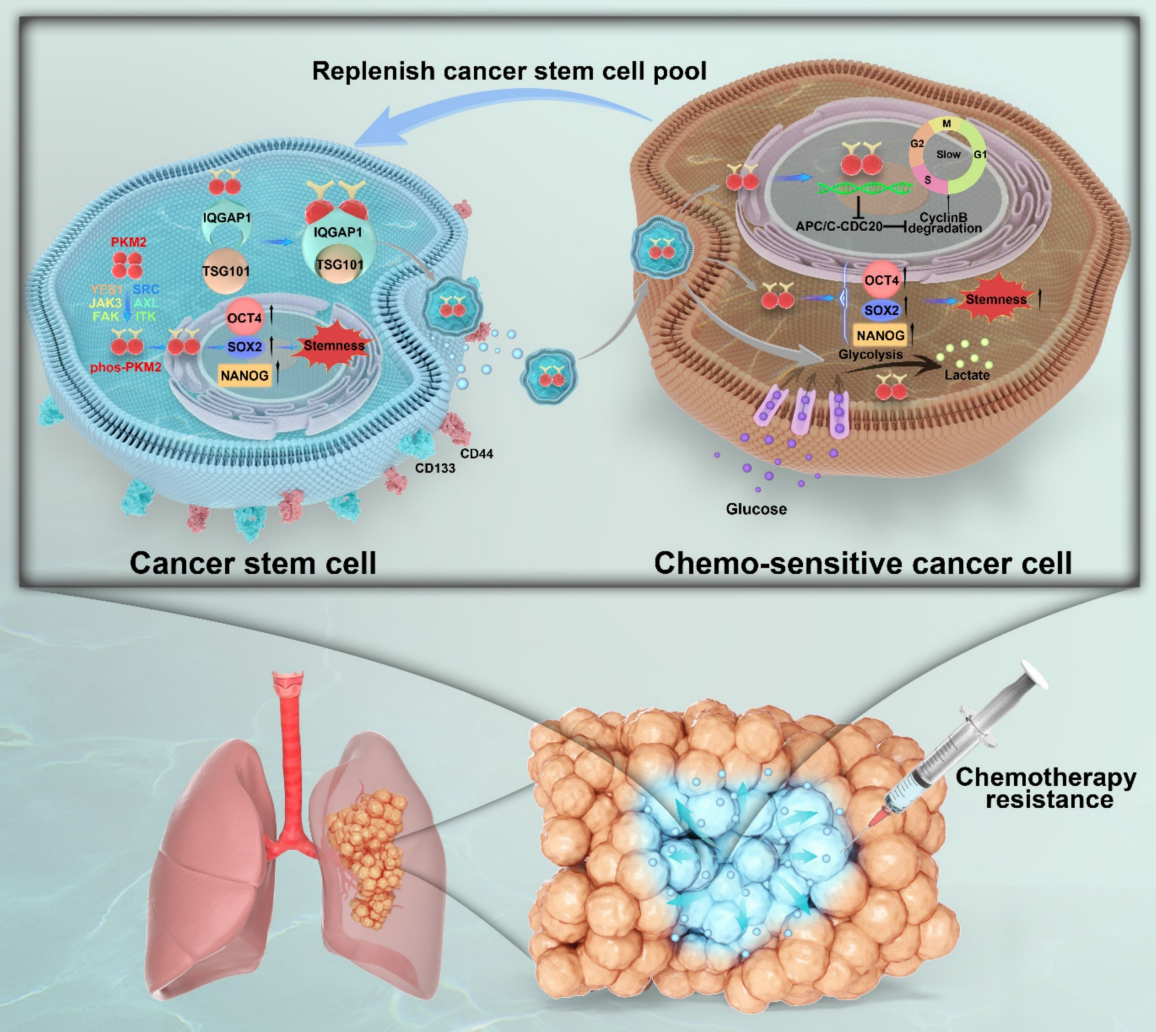
** Graphical summary of IQGAP1-mediated sorting of phos-PKM2 in CSC-derived sEVs and its role in chemoresistance in NSCLC.** This schematic illustrates the role of CSC-derived sEVs in promoting stemness and chemoresistance in NSCLC. In CSCs (left panel), surface markers CD44 and CD133 are expressed. Phosphorylation of PKM2 at Y105 (phos-PKM2) is induced by receptor tyrosine kinases (YES1, Src, JAK3, FAK, ITK, AXL), facilitating its transition from the tetrameric to the dimeric form. phos-PKM2 translocates to the nucleus to enhance the expression of stemness-related transcription factors (SOX2, NANOG, and OCT4). IQGAP1 mediates the selective incorporation of phos-PKM2 into sEVs through interactions with the ESCRT component TSG101, enabling the secretion of sEVs into the tumor microenvironment. In chemosensitive cancer cells (right panel), CSC-derived sEV uptake delivers phos-PKM2, inducing metabolic reprogramming (enhanced glycolysis and suppressed oxidative phosphorylation) and slowing the cell cycle via APC/C-CDC20 inhibition and reduced Cyclin B degradation. These processes collectively promote stemness and chemoresistance, replenishing the CSC pool. The lower section illustrates a lung tumor model, depicting how CSC-derived sEVs expand the CSC population within the tumor microenvironment, thereby driving chemotherapy resistance and malignant progression.

Unlocking phenotypic plasticity has recently been recognized as a dimension of cancer hallmarks 11. In this study, we explored the complex dynamics of CSCs and their capacity for phenotypic plasticity and illustrate the ability of non-CSCs to acquire or revert to CSC-like traits mediated by CSC-derived sEVs (Figure 9). This adaptability of CSCs is corroborated by ablation studies and lineage tracing in human colorectal cancer mouse models and organoid configurations, where research spearheaded by Shimokawa and Modrusan revealed that differentiated tumor cells could reacquire a LGR5+ state, indicative of colorectal cancer stem cell markers, and regain proliferative abilities following the targeted elimination of LGR5+ cells through cancer therapeutics or CRISPR editing techniques 13, 37. Ous is the first study to investigate the role of pY105-PKM2 as cargo within sEVs, highlighting its vital role in augmenting stemness characteristics in NSCLC recipient cells. Through this mechanism, pY105-PKM2 significantly contributes to the maintenance of the CSC pool and promotes therapeutic resistance.

sEVs harbor various functional proteins, RNAs, DNA fragments, and bioactives that orchestrate intercellular material transfer and information exchange, thereby regulating cellular functions and steering oncogenic processes 18, 38. There is also increasing evidence that sEVs promote cancer stemness 39, 40. Glioblastoma stem cells secrete sEVs that facilitate the formation of neurospheres and endothelial tubes and enhance glioblastoma invasiveness 41, and sEVs originating from fibroblasts augment colorectal CSC proliferation to bolster chemoresistance 42. Additionally, the tumorigenic capabilities of glioma stem-like cells are amplified through the transfer of miR-1587 by sEVs produced by glioma-associated mesenchymal stem cells 43. Our findings highlight the critical role of sEVs as mediators of intercellular communication within the CSC niche, revealing how CSC-derived sEVs facilitate interactions between CSCs and their normal counterparts. This, in turn, propagates stemness and chemoresistance and replenishes the CSC pool in NSCLC.

PKM2, a key rate-limiting enzyme of glycolysis, is overexpressed in various cancers and is instrumental in the metabolic reprogramming of cancer cells to prefer glycolysis for energy production, even in the presence of oxygen—a phenomenon known as the Warburg effect 44-46. The phosphorylation of PKM2 at Y105 is a key factor in sustaining the CSC state and facilitating tumor progression 31. We observed a correlation between elevated levels of pY105-PKM2 and poor prognosis in NSCLC patients, with the phosphorylated enzyme showing potential as a biomarker for disease progression and treatment outcomes. Most importantly, a strong association between the CSC marker CD133 and phosphorylated PKM2, but not its unmodified form, underscores that PKM2 phosphorylation at Y105 is a critical determinant of cancer cell stemness and drug resistance in NSCLC, and we confirmed this by constructing point mutation cells. Whether pY105-PKM2 can be sorted into sEVs, the underlying mechanisms, and the downstream effects on the behavior of recipient cells remain incompletely elucidated. However, our research shows that pY105-PKM2 is an essential sEV component, driving stemness in recipient non-CSCs and thereby supporting the CSC pool and enhancing chemoresistance. Notably, our study illuminates the selective sorting mechanism of pY105-PKM2 into sEVs mediated by the adaptor protein IQGAP1. This discovery links the phosphorylation state of a glycolytic enzyme to the sophisticated molecular sorting machinery within CSCs, highlighting a previously underappreciated layer of regulation in CSC propagation and sEV cargo specificity.

Cancer cell heterogeneity and plasticity are major barriers to the efficacy of current therapeutic strategies 47, 48. Within tumors, there exists a population of slow-cycling cells that are not actively proliferating and, hence, intrinsically resistant to treatments that target dividing cells 49-51. These slow-cycling cells share characteristics with CSCs, including quiescence, and can evade anti-neoplastic treatments, contributing to tumor relapse, maintaining tumor dormancy, and mediating metastasis ​^49^, ^52^, ^53^. In this study, the transfer of pY105-PKM2 via sEVs to recipient cells induced slower cell cycle progression, metabolic remodeling, and enhanced chemoresistance, highlighting the mechanisms underlying CSC resilience and the recurrence often seen in NSCLC. This study revealed that pY105-PKM2-induced changes in cell cycle dynamics in recipient cells mediated by the APC/C pathway may play a role in gaining stemness, although the exact molecular mechanisms need to be fully elucidated.

In conclusion, our study not only reinforces the central role of CSCs and their derived sEVs in NSCLC pathogenesis but also provides a mechanistic link between sEV-mediated intercellular communication and the propagation of stemness and drug resistance, emphasizing the importance of pY105-PKM2 in this process. By advancing our understanding of the molecular mechanisms driving CSC maintenance and therapeutic resistance, we pave the way for innovative treatment strategies targeting the sEV-mediated communication network in NSCLC.

## Supplementary Material

Supplementary figures and tables.
