# Supplementary material for: A novel pathway for stemness propagation and chemoresistance in non-small cell lung cancer via phosphorylated PKM2-loaded small extracellular vesicles
Source: Theranostics. 2025 Feb 24;15(8):3439–61. doi: 10.7150/thno.103722 (PMC11905138; doi:10.7150/thno.103722)
Supplement: Supplementary file 1 — Supplementary figures and tables. [file thnov15p3439s1.pdf]

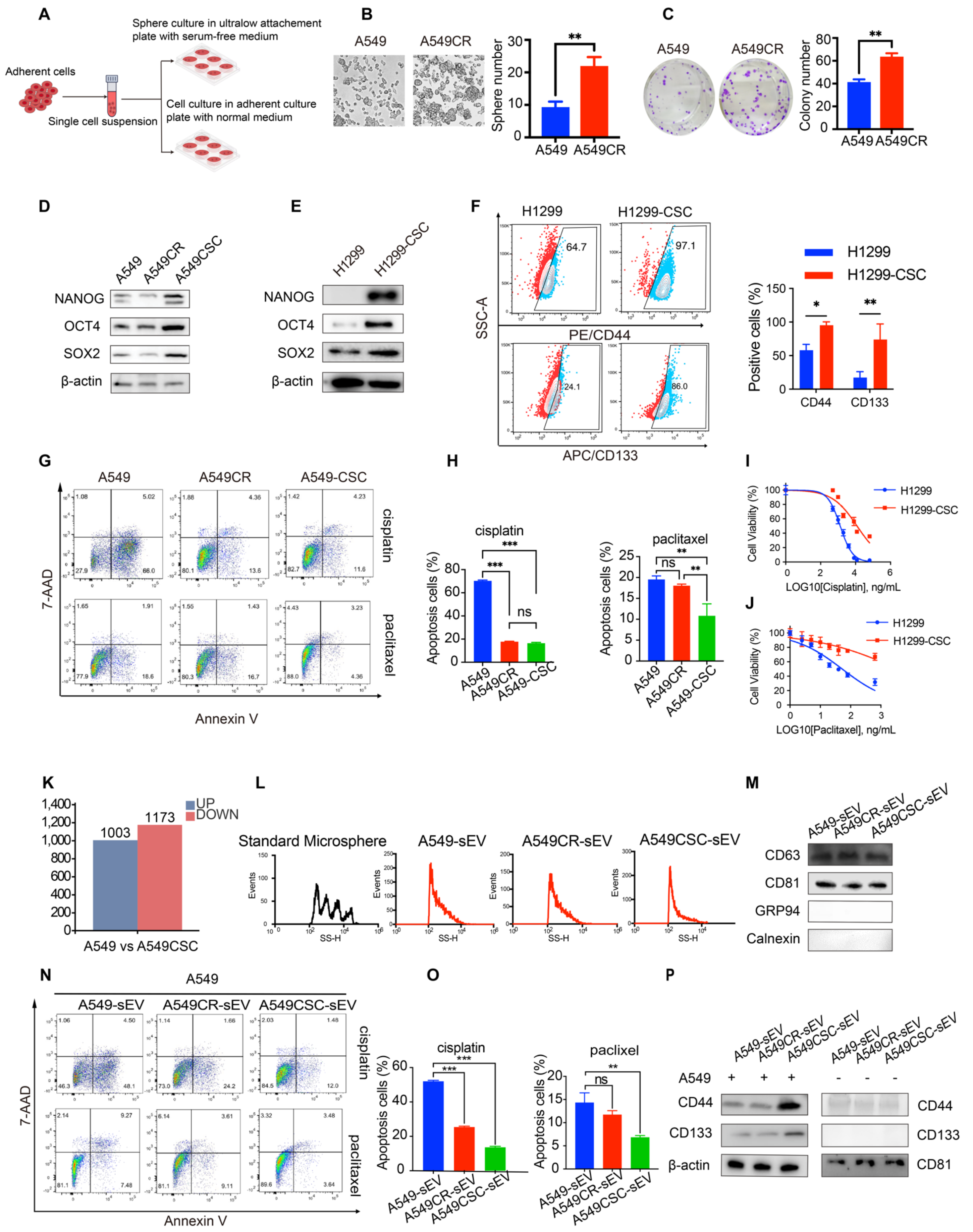

A
Sphere culture in ultralow attachement plate with serum-free medium
Adherent cells
Single cell suspension
Cell culture in adherent culture plate with normal medium
B
A549
A549CR
Sphere number
C
Colony number
D
A549 A549CR A549CSC
NANOG
OCT4
SOX2
β-actin
E
H1299 H1299-CSC
F
H1299
H1299-CSC
SSC-A
PE/CD44
APC/CD133
Positive cells (%)
CD44
CD133
G
A549
A549CR
A549-CSC
cisplatin
paclitaxel
7-AAD
Annexin V
H
Apoptosis cells (%)
I
Cell Viability (%)
LOG10[Cisplatin], ng/mL
J
LOG10[Paclitaxel], ng/mL
K
UP
DOWN
1003
1173
A549 vs A549CSC
L
Standard Microsphere
A549-sEV
A549CR-sEV
A549CSC-sEV
Events
SS-H
M
CD63
CD81
GRP94
Calnexin
N
O
paclixel
P

Figure S1: Characterization and impact of cancer stem cell-derived small extracellular vesicles (CSC-sEVs) on stemness and drug resistance.

(A) Schematic illustrating the sphere formation assay and colony formation assay. Single-cell suspensions from adherent cells were cultured in ultra-low attachment plates with serum-free medium to promote sphere formation or in normal medium for adherent culture. (B) Representative images and quantification of sphere formation in A549 and A549CR cells. Data are presented as mean ± SD (**$p < 0.01$, n = 3). (C) Representative images and quantification of colony formation in A549 and A549CR cells. Data are presented as mean ± SD (**$p < 0.01$, n = 3). (D, E) Western blot analysis showing expression levels of stem cell markers (NANOG, OCT4, SOX2) in A549, A549CR, and A549CSC populations (D), and in H1299 and H1299-CSC populations (E). (F) Flow cytometry analysis of CD44 and CD133 expression in H1299 and H1299-CSC populations. Quantification of CD44- and CD133-positive cells is shown as mean ± SD (*$p < 0.05$, **$p < 0.01$, n = 3). (G) Flow cytometry analysis of apoptosis in A549, A549CR, and A549CSC populations treated with cisplatin or paclitaxel (H) Quantification of apoptotic cells from (G). Data are presented as mean ± SD (ns: not significant, **$p < 0.01$, ***$p < 0.001$, n = 3). (I, J) IC50 curves showing cell viability of H1299 and H1299-CSC populations treated with cisplatin (I) and paclitaxel (J). Data are presented as mean ± SD (n = 3). (K) Differentially expressed proteins in A549CSC versus A549 cells identified based on a fold change threshold of ≥2 and an adjusted *p*-value of ≤0.05. (L) Representative nanoflow cytometry profiles of sEVs derived from A549, A549CR, and A549CSC cells, with standard microsphere size included as reference. (M) Western blot analysis of sEV markers (CD63, CD81) and negative markers (GRP94, Calnexin) in sEVs derived from A549, A549CR, and A549CSC cells. (N) Flow cytometry analysis of apoptosis in A549 cells treated with sEVs from A549, A549CR, and A549CSC cells (O) Quantification of apoptotic cells from (N) following cisplatin or paclitaxel treatment. Data are presented as mean ± SD (ns: not significant, **$p < 0.01$, ***$p < 0.001$, n = 3) (P) Western blot analysis of CD44 and CD133 expression in sEVs derived from A549, A549CR, and A549CSC cells and A549 cells treated with sEVs derived from different source.

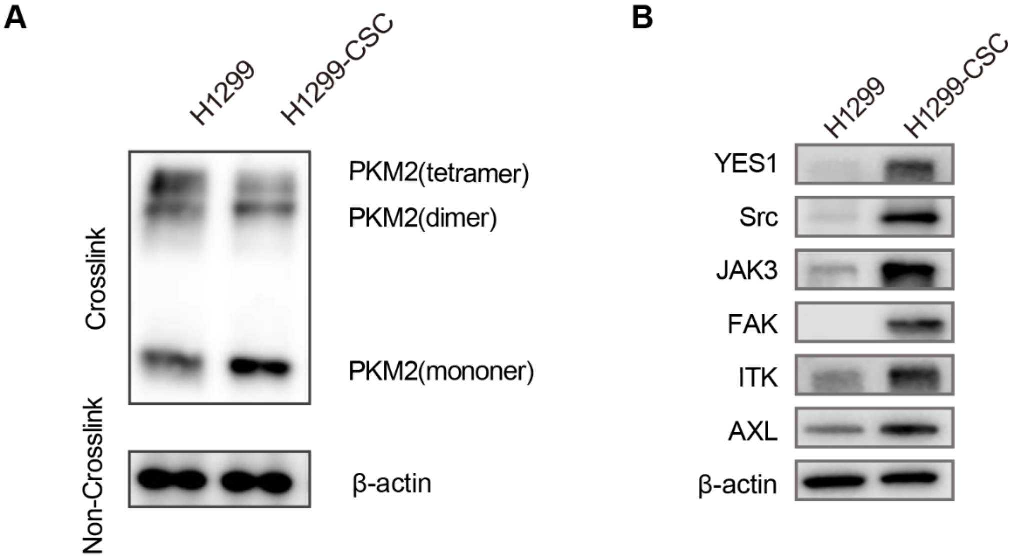

Figure S2. PKM2 configuration and tyrosine kinase expression in H1299 and H1299-CSC cells

(A) Western blot analysis showing the different oligomeric forms of PKM2 (tetramer, dimer, and monomer) in H1299 and H1299-CSC cells. (B) Western blot analysis of tyrosine kinases (YES1, Src, JAK3, FAK, ITK, and AXL) in H1299 and H1299-CSC cells.

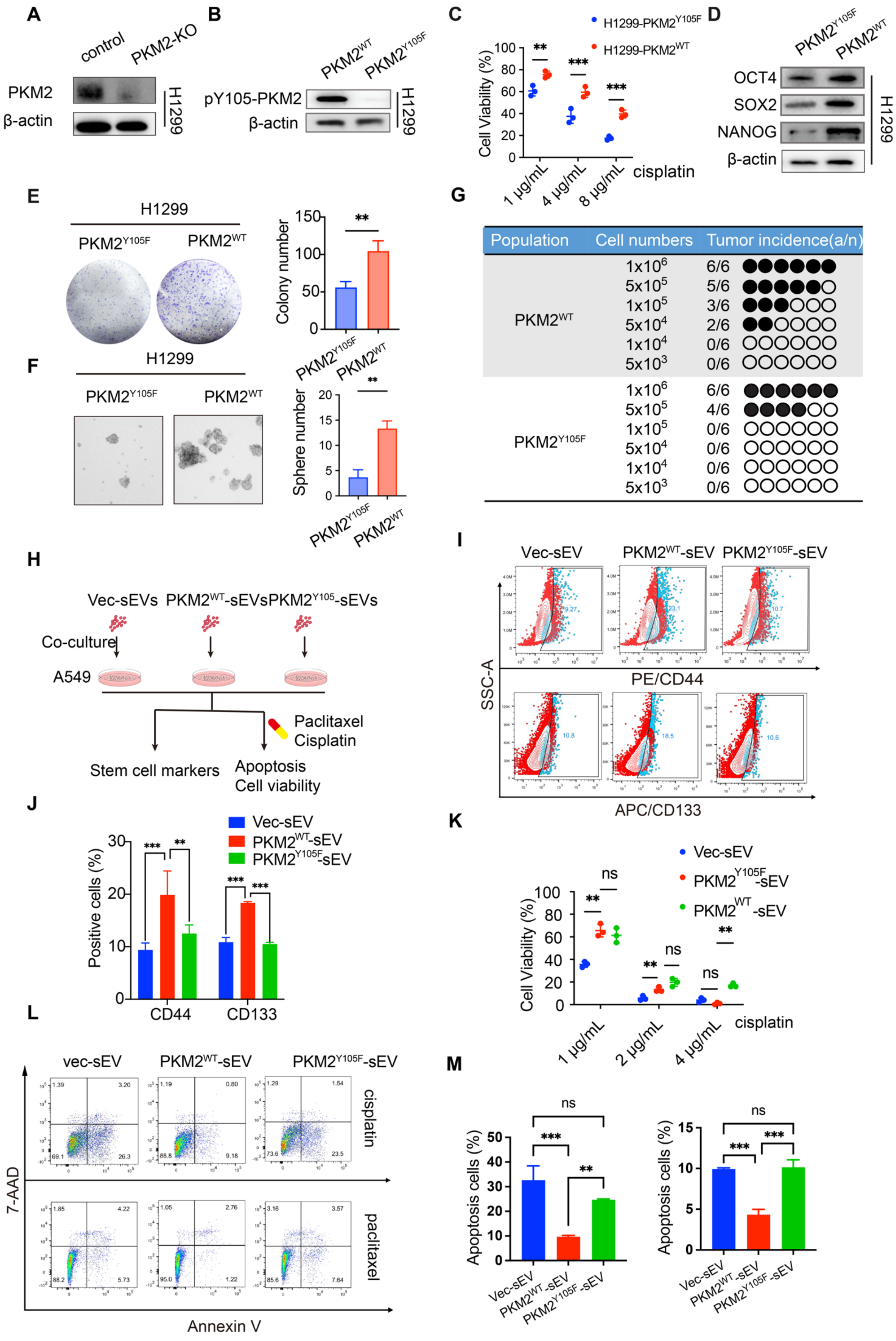
A
control
PKM2-KO
PKM2
β-actin
H1299
B
PKM2WT
PKM2Y105F
pY105-PKM2
β-actin
H1299
C
H1299-PKM2Y105F
H1299-PKM2WT
Cell Viability (%)
1 μg/mL
4 μg/mL
8 μg/mL
cisplatin
D
PKM2Y105F
PKM2WT
OCT4
SOX2
NANOG
β-actin
H1299
E
H1299
PKM2Y105F
PKM2WT
Colony number
F
H1299
PKM2Y105F
PKM2WT
Sphere number
G
Population
Cell numbers
Tumor incidence(a/n)
PKM2WT
1x10^6 6/6
5x10^5 5/6
1x10^5 3/6
5x10^4 2/6
1x10^4 0/6
5x10^3 0/6
PKM2Y105F
1x10^6 6/6
5x10^5 4/6
1x10^5 0/6
5x10^4 0/6
1x10^4 0/6
5x10^3 0/6
H
Vec-sEVs
PKM2WT-sEVs
PKM2Y105-sEVs
Co-culture
A549
Paclitaxel
Cisplatin
Stem cell markers
Apoptosis
Cell viability
I
Vec-sEV
PKM2WT-sEV
PKM2Y105F-sEV
SSC-A
PE/CD44
APC/CD133
J
Vec-sEV
PKM2WT-sEV
PKM2Y105F-sEV
Positive cells (%)
CD44
CD133
K
Vec-sEV
PKM2Y105F-sEV
PKM2WT-sEV
Cell Viability (%)
1 μg/mL
2 μg/mL
4 μg/mL
cisplatin
L
vec-sEV
PKM2WT-sEV
PKM2Y105F-sEV
cisplatin
paclitaxel
7-AAD
Annexin V
M
Apoptosis cells (%)
Vec-sEV
PKM2WT-sEV
PKM2Y105F-sEV

Figure S3. Experimental validation of $PKM2^{WT}$ and $PKM2^{Y105F}$ in non-small cell lung cancer (NSCLC) cells, and the effects of sEVs on recipient cells.

(A) Western blot analysis confirming PKM2 knockout in H1299 cells using CRISPR-Cas9. (B) Western blot showing the expression of pY105-PKM2 in $PKM2^{WT}$ and $PKM2^{Y105F}$ H1299 cells. (C) Cell viability assays of H1299-$PKM2^{WT}$ and H1299-$PKM2^{Y105F}$ cells treated with different concentrations of cisplatin. (D) Western blot analysis of OCT4, SOX2, and NANOG expression in $PKM2^{WT}$ and $PKM2^{Y105F}$ H1299 cells. (E) Representative images and quantified results of colony formation assays of H1299-$PKM2^{WT}$ and H1299-$PKM2^{Y105F}$ cells. (F) Representative images and quantified results of sphere formation assays of H1299-$PKM2^{WT}$ and H1299-$PKM2^{Y105F}$ cells. (G) Tumor incidence rates from limiting dilution assays in nude mice subcutaneously inoculated with different numbers of H1299-$PKM2^{WT}$ and H1299-$PKM2^{Y105F}$ cells. (H) Schematic representation of the experimental design for co-culture of chemosensitive A549 cells with sEVs derived from Vec-sEV, $PKM2^{WT}$-sEV, or $PKM2^{Y105F}$-sEV, followed by assessments of CD44 and CD133 expression, cell viability, and apoptosis. (I) Flow cytometry analysis showing CD44 and CD133 expression in A549 cells treated with Vec-sEV, $PKM2^{WT}$-sEV, or $PKM2^{Y105F}$-sEV. (J) Quantification of CD44- and CD133-positive cells in A549 cells treated with Vec-sEV, $PKM2^{WT}$-sEV, or $PKM2^{Y105F}$-sEV. (K) Cell viability assays of A549 cells treated with Vec-sEV, $PKM2^{WT}$-sEV, or $PKM2^{Y105F}$-sEV, followed by cisplatin or paclitaxel treatment. (L) Flow cytometry analysis of apoptosis in A549 cells treated with Vec-sEV, $PKM2^{WT-}$sEV, or $PKM2^{Y105F}$-sEV, followed by cisplatin or paclitaxel treatment. (M) Quantification of apoptotic cells (Annexin V-positive) in A549 cells treated with Vec-sEV, $PKM2^{WT}$-sEV, or $PKM2^{Y105F}$-sEV, followed by cisplatin or paclitaxel treatment. Data in (D), (E), (F), (J), (K), and (M) were analyzed by one-way ANOVA and are presented as mean ± SD. ns: not significant, $^{**}p < 0.01$, $^{***}p < 0.001$, n = 3.

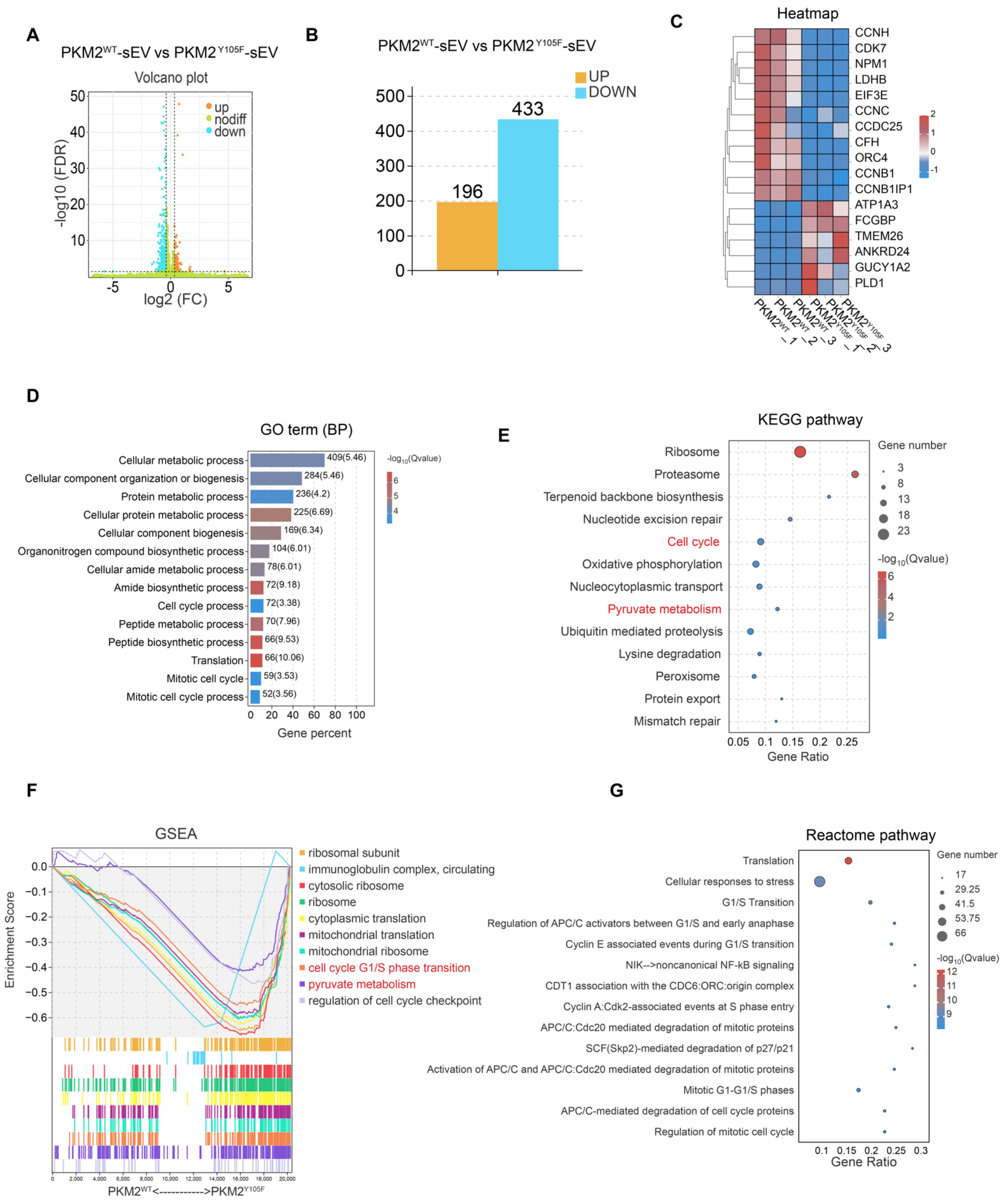
A
PKM2WT-sEV vs PKM2 Y105F-sEV
Volcano plot
up
nodiff
down
-log10 (FDR)
log2 (FC)
B
PKM2WT-sEV vs PKM2 Y105F-sEV
UP
DOWN
433
196
C
Heatmap
CCNH
CDK7
NPM1
LDHB
EIF3E
CCNC
CCDC25
CFH
ORC4
CCNB1
CCNB1IP1
ATP1A3
FCGBP
TMEM26
ANKRD24
GUCY1A2
PLD1
D
GO term (BP)
Cellular metabolic process 409(5.46)
Cellular component organization or biogenesis 284(5.46)
Protein metabolic process 236(4.2)
Cellular protein metabolic process 225(6.69)
Cellular component biogenesis 169(6.34)
Organonitrogen compound biosynthetic process 104(6.01)
Cellular amide metabolic process 78(6.01)
Amide biosynthetic process 72(9.18)
Cell cycle process 72(3.38)
Peptide metabolic process 70(7.96)
Peptide biosynthetic process 66(9.53)
Translation 66(10.06)
Mitotic cell cycle 59(3.53)
Mitotic cell cycle process 52(3.56)
Gene percent
-log10(Qvalue)
E
KEGG pathway
Ribosome
Proteasome
Terpenoid backbone biosynthesis
Nucleotide excision repair
Cell cycle
Oxidative phosphorylation
Nucleocytoplasmic transport
Pyruvate metabolism
Ubiquitin mediated proteolysis
Lysine degradation
Peroxisome
Protein export
Mismatch repair
Gene number
-log10(Qvalue)
Gene Ratio
F
GSEA
ribosomal subunit
immunoglobulin complex, circulating
cytosolic ribosome
ribosome
cytoplasmic translation
mitochondrial translation
mitochondrial ribosome
cell cycle G1/S phase transition
pyruvate metabolism
regulation of cell cycle checkpoint
Enrichment Score
PKM2WT<----------->PKM2Y105F
G
Reactome pathway
Translation
Cellular responses to stress
G1/S Transition
Regulation of APC/C activators between G1/S and early anaphase
Cyclin E associated events during G1/S transition
NIK-->noncanonical NF-kB signaling
CDT1 association with the CDC6:ORC:origin complex
Cyclin A:Cdk2-associated events at S phase entry
APC/C:Cdc20 mediated degradation of mitotic proteins
SCF(Skp2)-mediated degradation of p27/p21
Activation of APC/C and APC/C:Cdc20 mediated degradation of mitotic proteins
Mitotic G1-G1/S phases
APC/C-mediated degradation of cell cycle proteins
Regulation of mitotic cell cycle
Gene number
-log10(Qvalue)
Gene Ratio

Figure S4. Transcriptomic and pathway analysis of A549 cells treated with sEVs derived from A549-PKM2^{WT} and A549-PKM2^{Y105F}

(A) Volcano plot displaying differentially expressed genes (DEGs) between A549 cells treated with sEVs derived from PKM2^{WT} or PKM2^{Y105F} cells. DEGs were identified based on a fold-change threshold of ≥ 2 and adjusted $p \leq 0.01$. (B) Bar graph summarizing the number of upregulated and downregulated DEGs in A549 cells treated with PKM2^{WT}-sEVs compared to PKM2^{Y105F}-sEVs. (C) Heatmap representing DEGs selected based on transcriptomic analysis. (D) Gene Ontology (GO) biological process analysis of DEGs. (E) Kyoto Encyclopedia of Genes and Genomes (KEGG) pathway analysis of DEGs. (F) Gene Set Enrichment Analysis (GSEA) based on transcriptomic data. (G) Reactome pathway analysis of DEGs.

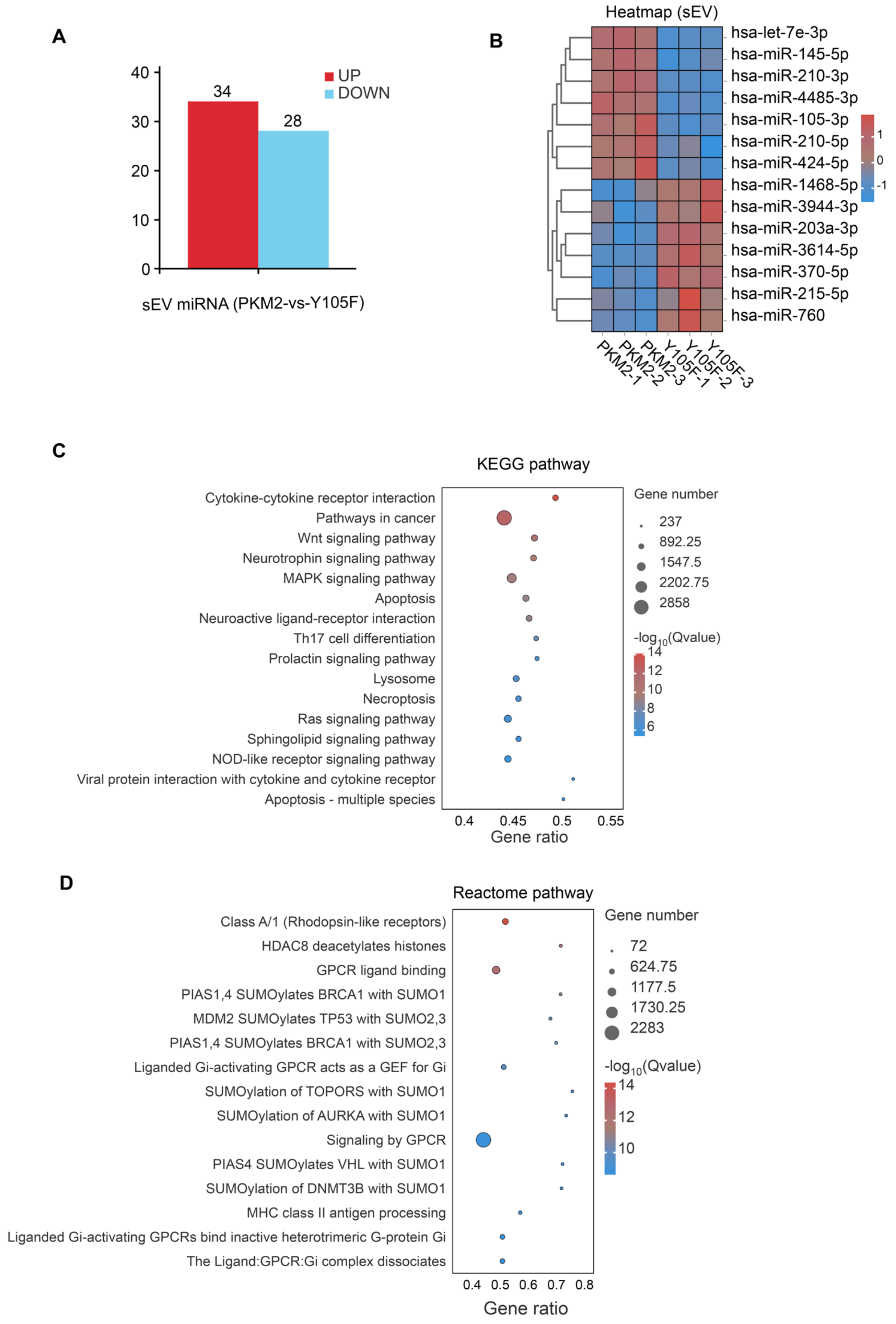
A
40
30
20
10
0
34
28
UP
DOWN
sEV miRNA (PKM2-vs-Y105F)
B
Heatmap (sEV)
hsa-let-7e-3p
hsa-miR-145-5p
hsa-miR-210-3p
hsa-miR-4485-3p
hsa-miR-105-3p
hsa-miR-210-5p
hsa-miR-424-5p
hsa-miR-1468-5p
hsa-miR-3944-3p
hsa-miR-203a-3p
hsa-miR-3614-5p
hsa-miR-370-5p
hsa-miR-215-5p
hsa-miR-760
1
0
-1
PKM2-1
PKM2-2
PKM2-3
Y105F-1
Y105F-2
Y105F-3
C
KEGG pathway
Cytokine-cytokine receptor interaction
Pathways in cancer
Wnt signaling pathway
Neurotrophin signaling pathway
MAPK signaling pathway
Apoptosis
Neuroactive ligand-receptor interaction
Th17 cell differentiation
Prolactin signaling pathway
Lysosome
Necroptosis
Ras signaling pathway
Sphingolipid signaling pathway
NOD-like receptor signaling pathway
Viral protein interaction with cytokine and cytokine receptor
Apoptosis - multiple species
0.4
0.45
0.5
0.55
Gene ratio
Gene number
237
892.25
1547.5
2202.75
2858
-log10(Qvalue)
14
12
10
8
6
D
Reactome pathway
Class A/1 (Rhodopsin-like receptors)
HDAC8 deacetylates histones
GPCR ligand binding
PIAS1,4 SUMOylates BRCA1 with SUMO1
MDM2 SUMOylates TP53 with SUMO2,3
PIAS1,4 SUMOylates BRCA1 with SUMO2,3
Liganded Gi-activating GPCR acts as a GEF for Gi
SUMOylation of TOPORS with SUMO1
SUMOylation of AURKA with SUMO1
Signaling by GPCR
PIAS4 SUMOylates VHL with SUMO1
SUMOylation of DNMT3B with SUMO1
MHC class II antigen processing
Liganded Gi-activating GPCRs bind inactive heterotrimeric G-protein Gi
The Ligand:GPCR:Gi complex dissociates
0.4
0.5
0.6
0.7
0.8
Gene ratio
Gene number
72
624.75
1177.5
1730.25
2283
-log10(Qvalue)
14
12
10

Figure S5. miRNA profiling of sEVs derived from A549-PKM2^{WT} and A549-PKM2^{Y105F} cells and pathway enrichment analysis

(A) Bar chart summarizing the number of upregulated and downregulated miRNAs identified in sEVs derived from PKM2^{WT} and PKM2^{Y105F} cells. (B) Heatmap displaying the expression profiles of selected differentially expressed miRNAs in sEVs derived from A549-PKM2^{WT} and A549-PKM2^{Y105F} cells. (C) KEGG pathway enrichment analysis of the predicted target genes of the differentially expressed miRNAs. (D) Reactome pathway enrichment analysis of the predicted target genes of the differentially expressed miRNAs.

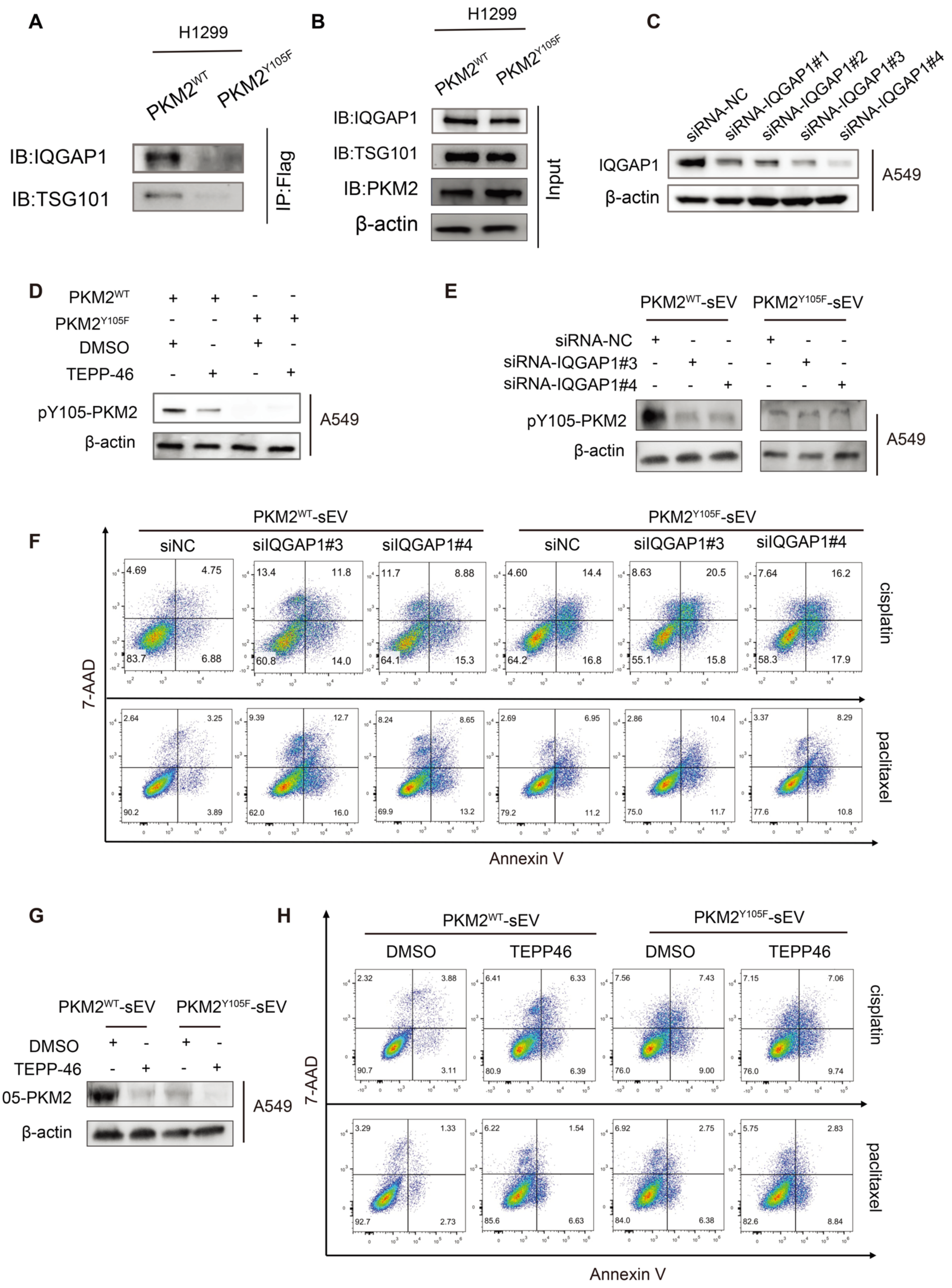
A
H1299
PKM2WT
PKM2Y105F
IB:IQGAP1
IB:TSG101
IP:Flag
B
H1299
PKM2WT
PKM2Y105F
IB:IQGAP1
IB:TSG101
IB:PKM2
β-actin
Input
C
siRNA-NC
siRNA-IQGAP1#1
siRNA-IQGAP1#2
siRNA-IQGAP1#3
siRNA-IQGAP1#4
IQGAP1
β-actin
A549
D
PKM2WT + + - -
PKM2Y105F - - + +
DMSO + - + -
TEPP-46 - + - +
pY105-PKM2
β-actin
A549
E
PKM2WT-sEV
PKM2Y105F-sEV
siRNA-NC + - - + - -
siRNA-IQGAP1#3 - + - - + -
siRNA-IQGAP1#4 - - + - - +
pY105-PKM2
β-actin
A549
F
PKM2WT-sEV
PKM2Y105F-sEV
siNC
siIQGAP1#3
siIQGAP1#4
siNC
siIQGAP1#3
siIQGAP1#4
4.69 4.75 83.7 6.88
13.4 11.8 60.8 14.0
11.7 8.88 64.1 15.3
4.60 14.4 64.2 16.8
8.63 20.5 55.1 15.8
7.64 16.2 58.3 17.9
cisplatin
2.64 3.25 90.2 3.89
9.39 12.7 62.0 16.0
8.24 8.65 69.9 13.2
2.69 6.95 79.2 11.2
2.86 10.4 75.0 11.7
3.37 8.29 77.6 10.8
paclitaxel
7-AAD
Annexin V
G
PKM2WT-sEV
PKM2Y105F-sEV
DMSO + - + -
TEPP-46 - + - +
pY105-PKM2
β-actin
A549
H
PKM2WT-sEV
PKM2Y105F-sEV
DMSO
TEPP46
DMSO
TEPP46
2.32 3.88 90.7 3.11
6.41 6.33 80.9 6.39
7.56 7.43 76.0 9.00
7.15 7.06 76.0 9.74
cisplatin
3.29 1.33 92.7 2.73
6.22 1.54 85.6 6.63
6.92 2.75 84.0 6.38
5.75 2.83 82.6 8.84
paclitaxel
7-AAD
Annexin V

Figure S6. IQGAP1 mediates the selective sorting of pY105-PKM2 into sEVs and influences drug resistance in recipient cells.

(A) Co-immunoprecipitation analysis of IQGAP1, TSG101, and PKM2 in H1299 cells stably expressing $PKM2^{WT}$. (B) Western blot analysis of IQGAP1, TSG101, and PKM2 in input lysates from H1299 cells expressing $PKM2^{WT}$ or $PKM2^{Y105F}$. (C) Validation of IQGAP1 knockdown efficiency in A549 cells using four independent siRNAs (siRNA-IQGAP1#1, siRNA-IQGAP1#2, siRNA-IQGAP1#3, and siRNA-IQGAP1#4) compared to the negative control (siRNA-NC) (D) Western blot analysis of pY105-PKM2 levels in A549-$PKM2^{WT}$ and A549-$PKM2^{Y105F}$ cells after treatment with TEPP-46 or DMSO. (E, G) Western blot analysis of pY105-PKM2 levels in A549 cells treated with sEVs derived from IQGAP1-silenced A549-$PKM2^{WT}$ cells(E) or TEPP-46-treated A549-$PKM2^{WT}$ cells(G). (F-H) Flow cytometry analysis of apoptosis in recipient A549 cells treated with sEVs derived from A549-$PKM2^{WT}$ or A549-$PKM2^{Y105F}$ cells following IQGAP1 silencing (F) or TEPP-46 treatment (H).

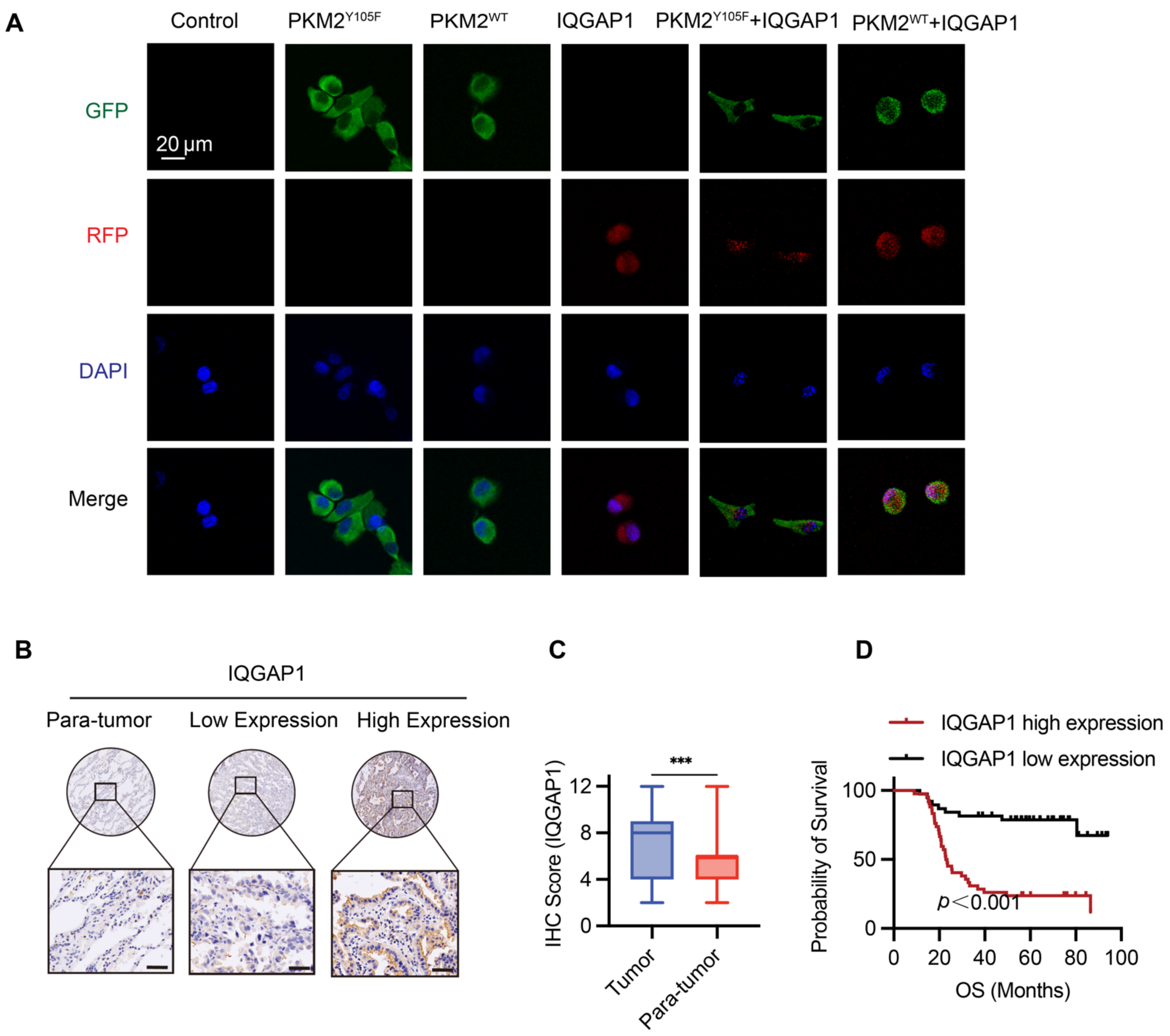

A
Control
PKM2Y105F
PKM2WT
IQGAP1
PKM2Y105F+IQGAP1
PKM2WT+IQGAP1
GFP
RFP
DAPI
Merge
20 μm
B
IQGAP1
Para-tumor
Low Expression
High Expression
C
IHC Score (IQGAP1)
***
0
4
8
12
Tumor
Para-tumor
D
IQGAP1 high expression
IQGAP1 low expression
Probability of Survival
100
50
0
p<0.001
0
20
40
60
80
100
OS (Months)

Figure S7. Validation of stable overexpression and clinical correlation of IQGAP1 and PKM2 in NSCLC.

(A) Immunofluorescence analysis of stable A549 cell lines overexpressing $PKM2^{WT}$ (green fluorescence), $PKM2^{Y105F}$ (green fluorescence), and/or IQGAP1 (red fluorescence). Nuclei were counterstained with DAPI (blue). Scale bar: 20 μm. (B) Representative immunohistochemical images of IQGAP1 expression in para-tumor and tumor tissues. Tumors were further categorized into low and high IQGAP1 expression groups. Scale bar: 50 μm. (C) Quantification of immunohistochemical staining showing IQGAP1 expression levels in tumor versus para-tumor tissues. Data are presented as mean ± SD, ***$p < 0.001$ (n = 80). (D) Kaplan-Meier survival analysis comparing overall survival (OS) between patients with low and high IQGAP1 expression levels. Log-rank test, $p < 0.001$.

Table S1. Correlation between PKM2 and clinicopathologic characteristics of NSCLC

		**ALL CASES**	**PKM2 HIGH**	**PKM2 LOW**	***p*-VALUE**
Participants		80	57	23	
Sex	Male	47	36	11	0.207
	Female	33	21	12	
Age	＜60 years	29	20	9	0.734
	≥60 years	51	37	14	
Tumor metastasis	Yes	20	16	4	0.318
	No	60	41	19	
Lymph node metastasis	Yes	22	20	2	0.017*
	No	58	37	21	
Relapse after chemotherapy	Yes	40	24	16	0.026*
	No	40	33	7	
TNM classification	I	28	15	13	0.025*
	II	12	8	4	
	III	24	22	2	
	IV	16	12	4	
Tumor diameter	＜3cm	36	25	11	0.747
	≥3cm	44	32	12	
Serum CYFRA 21-1	＜5ng/mL	8	4	4	0.057
	5-30 ng/mL	39	25	14	
	＞30 ng/mL	33	28	5	
Serum CEA	＜5ng/mL	15	10	5	0.233
	5-30 ng/mL	44	29	15	
	＞30 ng/mL	21	18	3	

*Significantly different ($p < 0.05$).

Table S2. Correlation between pY105-PKM2 and clinicopathologic characteristics of NSCLC

		ALL CASES	pY105-PKM2 HIGH	pY105-PKM2 LOW	*p*-VALUE
Participants		80	56	24	
Sex	Male	47	34	13	0.586
	Female	33	22	11	
Age	＜60 years	29	21	8	0.722
	≥60 years	51	35	16	
Tumor metastasis	Yes	20	15	5	0.573
	No	60	41	19	
Lymph node metastasis	Yes	22	20	2	0.012*
	No	58	36	22	
Relapse after chemotherapy	Yes	40	33	7	0.015*
	No	40	23	17	
TNM classification	I	28	15	13	0.007*
	II	12	7	5	
	III	24	23	1	
	IV	16	11	5	
Tumor diameter	＜3cm	36	24	12	0.556
	≥3cm	44	32	12	
Serum CYFRA 21-1	＜5ng/mL	8	5	3	0.843
	5-30 ng/mL	39	27	12	
	＞30 ng/mL	33	24	9	
Serum CEA	＜5ng/mL	15	10	5	0.443
	5-30 ng/mL	44	29	15	
	＞30 ng/mL	21	17	4	

*Significantly different ($p < 0.05$).

Table S3. Correlation between IQGAP1 and clinicopathologic characteristics of NSCLC

		**ALL CASES**	**IQGAP1 HIGH**	**IQGAP1 LOW**	***p*-VALUE**
Participants	Total	80	42	38	
Sex	Male	47	29	18	0.049*
	Female	33	13	20	
Age	＜60 years	29	14	15	0.568
	≥60 years	51	28	23	
Tumor metastasis	Yes	20	15	5	0.020*
	No	60	27	33	
Lymph node metastasis	Yes	22	15	7	0.084
	No	58	27	31	
Relapse after chemotherapy	Yes	40	30	10	＜0.001*
	No	40	12	28	
TNM classification	I	28	7	21	＜0.001*
	II	12	4	8	
	III	24	20	4	
	IV	16	11	5	
Tumor diameter	＜3cm	36	16	20	0.192
	≥3cm	44	26	18	
Serum CYFRA 21-1	＜5ng/mL	8	3	5	0.224
	5-30 ng/mL	39	18	21	
	＞30 ng/mL	33	21	12	

*Significantly different ($p < 0.05$).
